# Engineering Renewable Lignocellulosic Biomass as Sustainable Solar-Driven Interfacial Evaporators

**DOI:** 10.1007/s40820-025-02000-y

**Published:** 2026-01-05

**Authors:** Jinlong Zhu, Jifei Zhang, Jincheng Zha, Siqi Zhao, Wenfeng Ren, Bing Wang, Ling-Ping Xiao, Sanwei Hao, Changyou Shao, Jun Yang, Runcang Sun

**Affiliations:** 1 Liaoning Key Laboratory of Lignocellulose Chemistry and BioMaterials, Liaoning Collaborative Innovation Center for Lignocellulosic Biorefinery, College of Light Industry and Chemical Engineering, Dalian Polytechnic University, Dalian, 116034 People’s Republic of China; 2 School of Materials Science and Engineering, Shandong University of Technology, Zibo, 255000 People’s Republic of China; 3 Beijing Key Laboratory of Lignocellulosic Chemistry College of Materials Science and Technology, Beijing Forestry University, Beijing, 100083 People’s Republic of China

**Keywords:** Lignocellulosic biomass, Wood, Cellulose, Lignin, Solar-driven interfacial evaporators

## Abstract

The increasing scarcity of freshwater resources has driven the rapid emergence of solar-driven interfacial evaporators (SDIEs) as a sustainable approach to harvest fresh water by utilizing solar energy. Lignocellulosic biomass, featuring natural abundance, excellent renewability, unique natural structures, and superior biodegradability compared to the synthetic polymers, is highly attractive for constructing solar steam generators. This review aims to offer an innovative and in-depth insight into designing and optimizing high-performance integrated solar interfacial evaporators derived from renewable lignocellulosic biomass. First, the structural characteristics of lignocellulosic biomass are briefly introduced, serving as photothermal layer or supporting substrates in SDIEs. Secondly, the fabrication methods and processing technologies of lignocellulosic biomass-based evaporators are summarized from the perspective of photothermal layer and supporting substrates. Next, the most recent advances of regulation and optimization strategies are proposed to improve evaporation efficiency. Subsequently, this review summarizes the diverse functionalities of SDIEs, including desalination, power generation, wastewater treatment and antimicrobial, atmospheric water harvesting, and photocatalytic hydrogen production. Finally, the challenges in this field and outlook on the future development are discussed, which are anticipated to provide new opportunities for the advancement of lignocellulosic biomass-based SDIEs.

## Highlights

- This review systematically summarizes solar evaporator design and optimization using renewable lignocellulosic biomass.
- Unique structural merits and fabrication methods for photothermal layer and hydrophilic substrate are thoroughly discussed.
- Multifunctional integrated applications beyond desalination are highlighted.
- Current challenges and future development opportunities for scalable biomass-based evaporators are outlined.

## Graphical abstract

**Figure:**
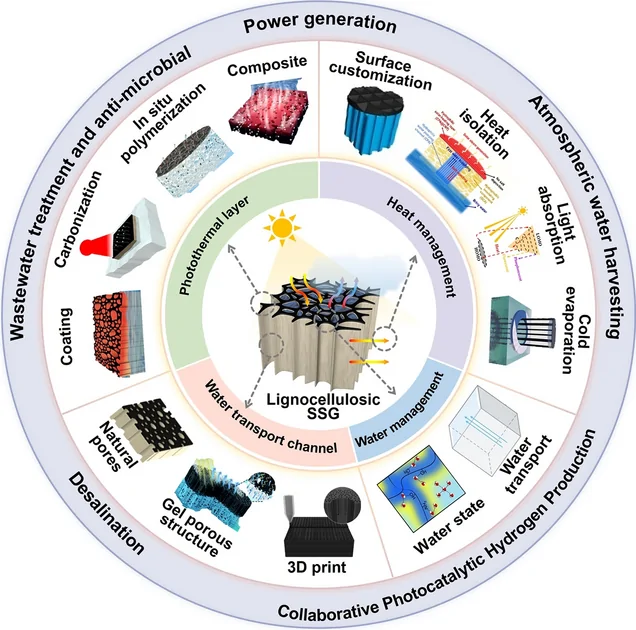

## Introduction

Water scarcity, one of the most strenuous global challenges today, arises from the limited availability of freshwater, with nearly half of the population of world facing severe shortages [14]. Despite water covering approximately three-quarters of the Earth surface, only ~ 2.5% is freshwater, with the remaining 97.5% consisting of saline water [15]. This imbalance has driven increasing attention toward desalination technologies as a potential solution to alleviate freshwater shortages. To address this issue, numerous desalination techniques have been employed to generate clean freshwater from seawater [16], frequently including reverse osmosis [17], electrodialysis [18], freezing [19], and multi-stage flash [20]. While effective and reliable, these methods rely on auxiliary equipment and secondary energy sources, such as thermal or electrical energy predominantly derived from fossil fuels or other nonrenewable resources. In contrast, solar energy has emerged as a highly attractive alternative due to its renewable nature and environmental sustainability, effectively addressing the escalating global demand for low-carbon energy solutions [21]. Among various solar-driven desalination technologies, solar-driven interfacial evaporators (SDIEs) have attracted particular interest for its ability to concentrate heat for localized evaporation of small water volumes, ensuring efficient utilization of solar energy.

The conventional bilayered SDIE architecture combines a photothermal conversion layer with a porous water transport substrate, simultaneously ensuring efficient solar absorption, continuous water supply, and thermal insulation for sustained vapor production. Substantial research has advanced photothermal layers with broad-spectrum absorption to optimize conversion efficiency, alongside engineered substrates that ensure effective thermal regulation and unhindered water transport to enhance SDIEs performance [22]. However, the traditional photothermal materials, including noble metal nanomaterials [23], transition metal materials [24, 25], carbon-based nanomaterials [26], organic conjugated materials [27], applied in the photothermal layers of interfacial evaporators, are often limited by their narrow light absorption spectra, high costs, and poor biocompatibility. With regard to supporting substrates in SDIEs, extensively utilized petroleum-derived synthetic polymers, such as polyurethane (PU) [28], and polystyrene (PS) [29], suffer from limited raw material availability, nonrenewability, nonbiodegradability, and inefficient recycling. Thus, the growing urgency to mitigate environmental degradation and fossil fuel reliance is accelerating significant demand for renewable and sustainable bio-based alternatives to replace conventional photothermal materials and supporting substrates for constructing SDIEs.

**Fig. 1:**
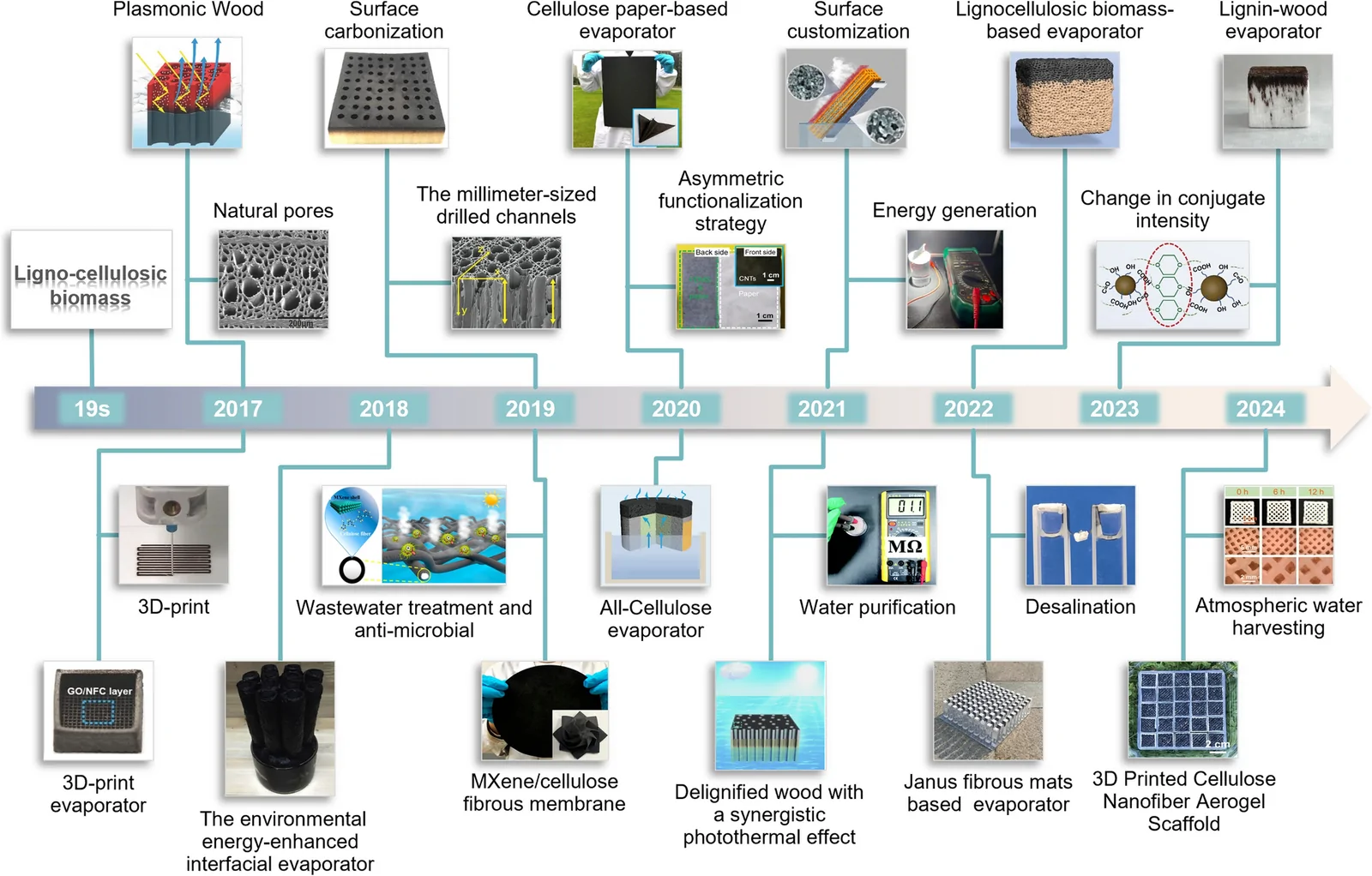
Brief timeline of lignocellulosic biomass for SDIE. Plasmonic wood-enabled high-efficiency interfacial solar evaporation [1]. Monolithic 3D-printed SDIE [2]. Ambient energy-enhanced SDIE [3]. A high-performance wood-based SDIE for continuous water desalination [4]. Anti-fouling MXene-cellulose fibrous membranes for sustainable solar water purification [5]. Asymmetric functionalization drives simultaneous clean water and electricity generation [6]. All cellulose-based SDIE with self-powered water wave detection [7]. Asymmetrically structured evaporator realizes efficient concurrent water and electricity production [8]. Cellulose-based SDIE with synergetic photothermal effect for optimized electricity generation and desalination [9]. All-lignocellulose biporous hydrogel architectures for solar evaporation [10]. Salt-resistant solar desalination and mineral recovery via suspended Janus fibrous membrane evaporator [11]. Lignin-functionalized wood evaporator for high-performance solar-powered water purification [12]. 3D-printed cellulose nanofiber scaffolds with multi-scale porosity for sustainable atmospheric moisture harvesting [13]

Lignocellulosic biomass as the largest reserves of renewable resources in nature attracts significant attention owing to its renewability, biocompatibility, and potential to address sustainability challenges, rendering it an ideal candidate for constructing SDIEs compared to the synthetic polymers. Wood, a typical example of lignocellulosic biomass, is regarded as a sustainable structural material. Its inherent multilayered and porous structure reminiscent of eggshell membranes demonstrates superior thermal insulation and natural hydrophilicity, which are exceptionally well-suited both as a thermal insulator and water conduit in solar interfacial evaporators. Notably, the natural advantages of wood with inherent efficient water transport and heat insulation enable to simplify device design, reduce production costs, and support scalability in desalination applications [30]. Cellulose and lignin, the primary structural constituents of wood, have been widely investigated for use in SDIEs. Cellulose exhibits excellent hydrophilicity, high mechanical strength, axial rigidity and modulus, structural stability, and chemical reactivity [31], which render cellulose-based substrates highly adaptable for SDIEs applications and hold significant promise for sustainable development. Meanwhile, lignin is distinguished by its highly intricate molecular architecture, which encompasses a diverse array of functional groups, such as hydroxyl, carboxyl, and epoxy moieties, thereby enabling a broad range of chemical modifications [32]. Notably, the strong polycyclic *π*-conjugated framework of lignin promotes *π*–*π* molecular interactions, which are instrumental for efficient and sustainable photothermal conversion [33]. Figure 1 presents a chronological analysis of key developments in lignocellulosic biomass-based SDIEs, tracking the evolution from initial wood-structured systems to contemporary lignocellulosic biomass-based SDIEs. The timeline highlights annually emerging designs that demonstrate innovative approaches to either structural engineering or functional application, revealing both the dynamic progression and substantial future potential of this research domain.

**Fig. 2:**
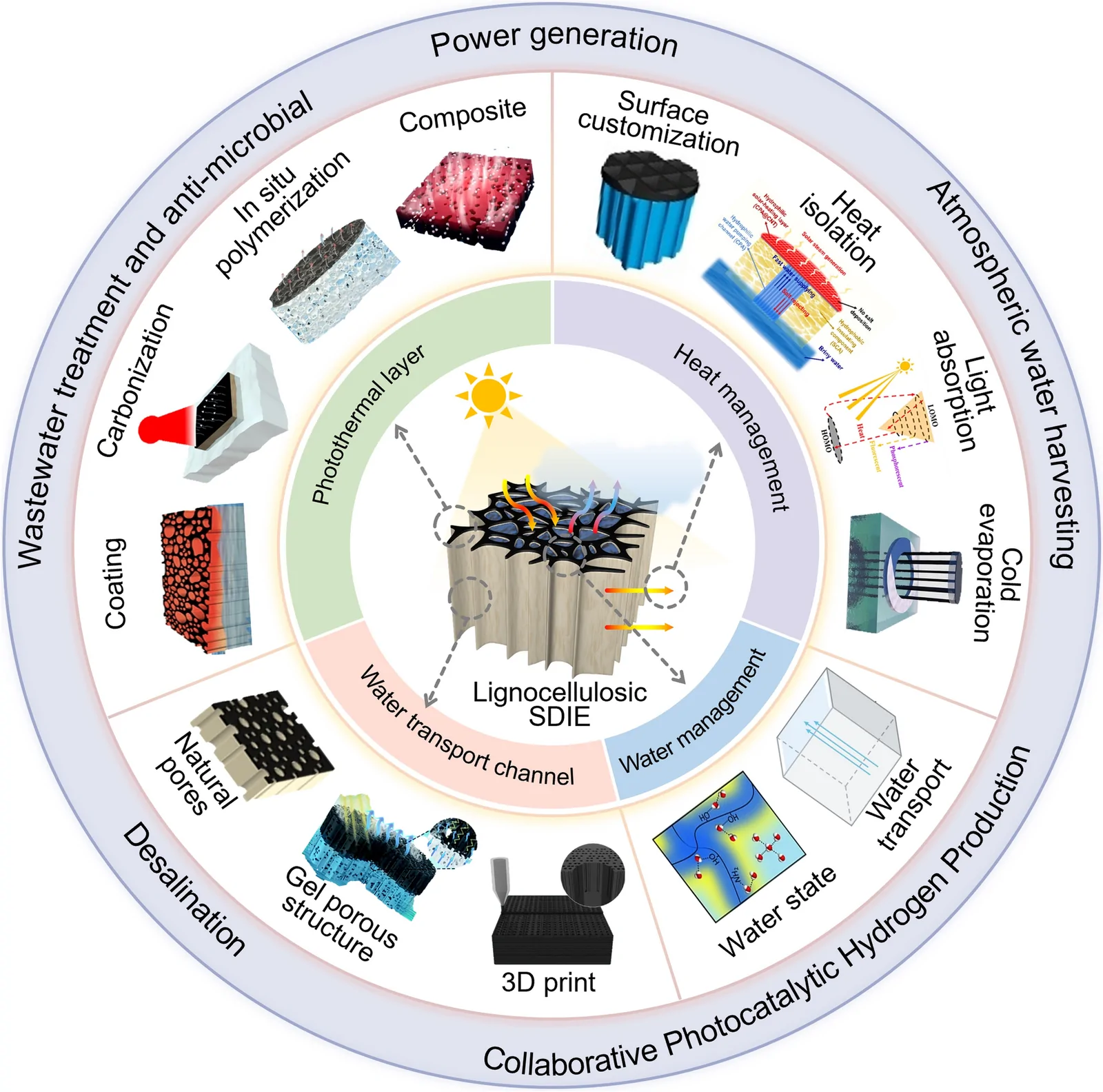
Schematic showing the design and application of the lignocellulosic biomass-based SDIEs. Coating [34], carbonization [35], in situ polymerization [36], composite [37], surface customization [38], heat isolation [39], light absorption [40], cold evaporation [41], water transport [42], water state [43], 3d print [44], gel porous structure [45], nature pores [46]

Previous reviews of lignocellulosic biomass-based SDIEs have predominantly focused on cellulose- or wood-based systems in isolation, neglecting the unique intrinsic photothermal properties of lignin, a key biomass component, which could be integrated as a natural photothermal material. Additionally, the optimization and regulation of biomass-based SDIEs have been absent, with limited attention given to their potential integration between solar evaporation and other applications. This review systematically summarizes the recent progress in design strategies, optimization methodologies, and multifunctional integrated applications of interfacial evaporators derived from wood, cellulose, and lignin, as illustrated in Fig. 2. Through structural analysis of wood, cellulose, and lignin, we highlight the design strategies and management measures for SDIEs. Then, we conclude the multifunctional integration of lignocellulosic biomass-based SDIEs for engineering applications of desalination, power generation, wastewater treatment and antimicrobial, atmospheric water harvesting, and photocatalytic hydrogen production. Finally, we discuss the scientific and technological challenges and potential opportunities and provide comprehensive guidance on the design, optimization, and application of lignocellulosic biomass-based SDIEs.

## Lignocellulosic Biomass

Lignocellulosic biomass, the most abundant renewable organic resource on Earth, is composed of cellulose, hemicellulose, and lignin, which collectively form the structural skeleton of plant cell walls [47]. From a bottom-up perspective, lignin and cellulose offer distinct functional advantages. Specifically, the innate photothermal properties of lignin and the pronounced hydrophilicity of cellulose, render them particularly suitable for integration into evaporative systems. From a top-down perspective, natural wood provides a natural matrix of microchannels that facilitates efficient water transport. Consequently, this review systematically examines and summarizes the applications of wood, cellulose, and lignin, reflecting the current research emphases and technological pathways in the field.

### Wood

**Fig. 3:**
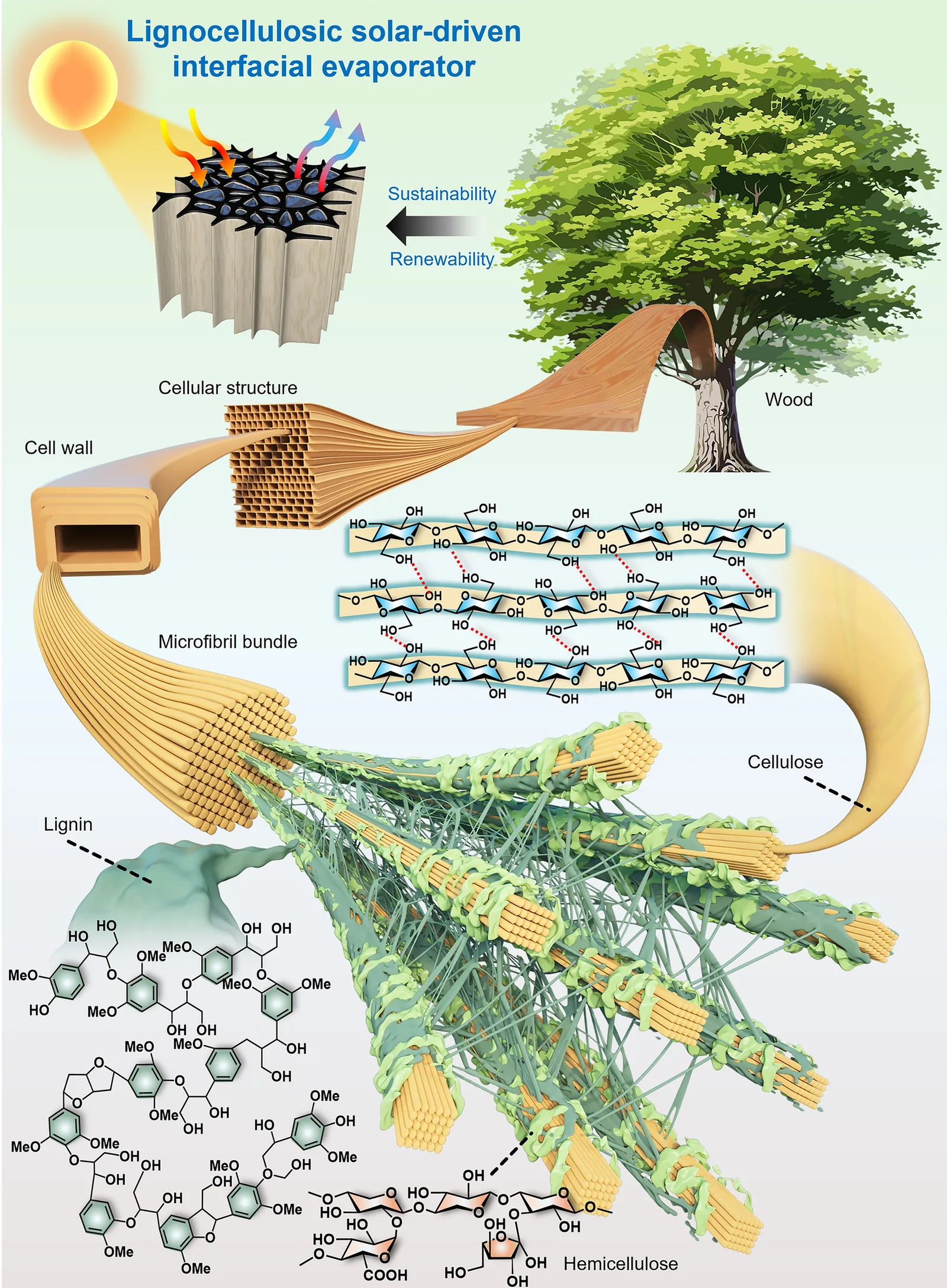
Schematic illustration of hierarchical structure of lignocellulosic biomass. (Color figure online)

Wood, the largest renewable biomass resource, consists of three main components: cellulose, hemicellulose, and lignin (Fig. 3) [48]. Cellulose, constituting 40–50% of the wood, serves as the primary structural element, with its crystalline regions providing strength, akin to a skeletal framework [49]. Hemicellulose (10–30%), functioning as a filler, occupies the interstitial spaces between cellulose microfibrils and is cohesively bound to cellulose by lignin (20–30%), which serves as a natural adhesive within the cell wall matrix. The proportions of three components vary across the wood species, with softwood largely composed of parenchyma and tracheids, while hardwood exhibits a more intricate microstructure that includes vessels, fibrous elements, and parenchyma (with tracheids present in some hardwood species) [50, 51]. These wood cells, which differ in shape, size, and arrangement, are densely packed to form the unique multi-layered porous structure of wood. Wood exhibits a hierarchical void architecture spanning three distinct size regimes: macrovoids, microvoids, and mesovoids. Macrovoids, which are visible to the naked eye, are constituted by wood cells (ranging from 50 to 1500 µm in width and 0.1 to 10 mm in length), vessels (20 to 400 µm), tracheids (15 to 40 µm), and intercellular spaces (50 to 300 µm) [52]. This naturally occurring multi-layered, porous architecture, resembling that of an eggshell membrane, imparts wood with remarkable thermal insulation properties.

Tree trunks serve as primary as conduits for the transport of nutrients and water, facilitated by aligned, growth-oriented channels within the wood. These channels, which contain microporous pits, enable efficient material exchange. The presence of these growth-aligned directional channels imparts wood with a range of anisotropic properties, predominantly its remarkable capacity for directional water transport. Notably, the anisotropic thermal conductivity of wood significantly enhances its thermal insulation performance [53, 54], rendering it an effective substrate for interfacial solar evaporators. As a result, wood fulfills all critical roles in three essential components of SDIEs: photothermal layer, thermal insulation, and water channels. Benefitted from inherent dual functionality in water transport and thermal insulation, wood enables to simplify equipment design, thereby reducing manufacturing costs, and promotes and facilitates scalable implementation of desalination technologies.

### Cellulose

As the most abundant renewable biopolymer on Earth, cellulose is primarily derived from plant-based sources, including fungi, trees, algae, annual plants, and bacteria, with plant fibers serving as the predominant reservoir [55]. As a fundamental structural component, it reinforces the mechanical integrity of wood and other plant tissues. While certain sources, in cotton seed hairs, contain cellulose in highly pure forms exceeding 90 wt%, it more commonly occurs in a composite structure alongside lignin, hemicelluloses, pectin, and trace organic compounds [56]. Industrially, cellulose is largely extracted from wood pulp, which remains a key raw material for various applications [57].

As a linear polysaccharide, cellulose is a homopolymer composed of thousands of β-1,4-linked d-glucose units, with cellobiose as its repeating dimeric unit. The degree of cellulose polymerization (ranging from 300 to 16,000) exhibits significant variation across source materials [55, 58]. Interchain hydrogen bonding (denoted by red dotted lines in Fig. 3) induces cellulose chain stacking, generating elementary fibrils that subsequently aggregate into microfibrils. Concurrently, intrachain hydrogen bonds between hydroxyl groups and adjacent ring oxygens stabilize the molecular structure, preserving linear chain conformation of cellulose. Additionally, the van der Waals and intermolecular hydrogen bonding collectively drive the parallel stacking of cellulose chains, contributing to the formation of fibrillar structures. These intra- and intermolecular interactions render cellulose a structurally rigid and thermally stable polymer. Its linear structures and extensive hydrogen bonding confer not only mechanical stiffness but also key properties, including biocompatibility, degradability, hydrophilicity, high strength, high thermal stability, and durability [48, 57, 58].

### Lignin

Lignin is the most abundant aromatic biopolymers in nature [59–62]. In contrast to cellulose, lignin is a complex, amorphous three-dimensional polymer composed of oxygenated p-propylphenol units. It plays a crucial role in reinforcing structural rigidity of the plant cell wall [63], while also providing resistance to microbial attack [64]. The primary monomeric constituents of lignin include p-hydroxyphenyl (H), guaiacyl (G), and syringyl (S) [65], which are interconnected through diverse intricate linkages, such as β-O-4 and β-5 linkages. The molecular architecture of lignin is further characterized by a rich array of functional groups, including carboxyl, carbonyl, and hydroxyl moieties, which significantly contribute to its diverse chemical reactivity [66–68]. These structural and chemical features impart a broad spectrum of remarkable properties to lignin, such as ultraviolet (UV) shielding [69, 70], anti-aging [71, 72], and excellent adsorption and dispersion capabilities [73]. Moreover, the number and positioning of methoxy groups on the phenolic rings of lignin monomers exhibit considerable variability, which further modulates its functional properties. The distribution of these monomeric units is not uniform and varies significantly across different plant species, a factor that plays a critical role in determining lignin functionality and its potential applications across diverse fields.

Notably, structural framework of lignin comprises multiple benzene rings, which facilitate electron delocalization across the polymer. This delocalized distribution of electrons creates an "electron cloud," endowing the benzene ring with a highly stable conjugated system. Strong conjugated systems and *π*–*π* stacking in lignin generate distinctive optical properties [74, 75]. Upon absorbing light energy, *π* electrons in lignin can transition from the valence band to the conduction band, resulting in the formation of excited-state electrons. The excited electrons undergo nonradiative relaxation to their ground state, with the energy released during this process manifested as heat [76]. Simultaneously, the hydroxyl groups of lignin form dynamic noncovalent bonds with water molecules, modulating both aqueous phase organization and intermediate water content through these interfacial interactions [77]. This interaction enhances the evaporation performance of the material, while the inherent low thermal conductivity of lignin provides exceptional thermal insulation to minimize heat loss, collectively offering a promising prospect for designing sustainable SDIEs utilizing lignin [78].

## Construction of SDIEs

**Fig. 4:**
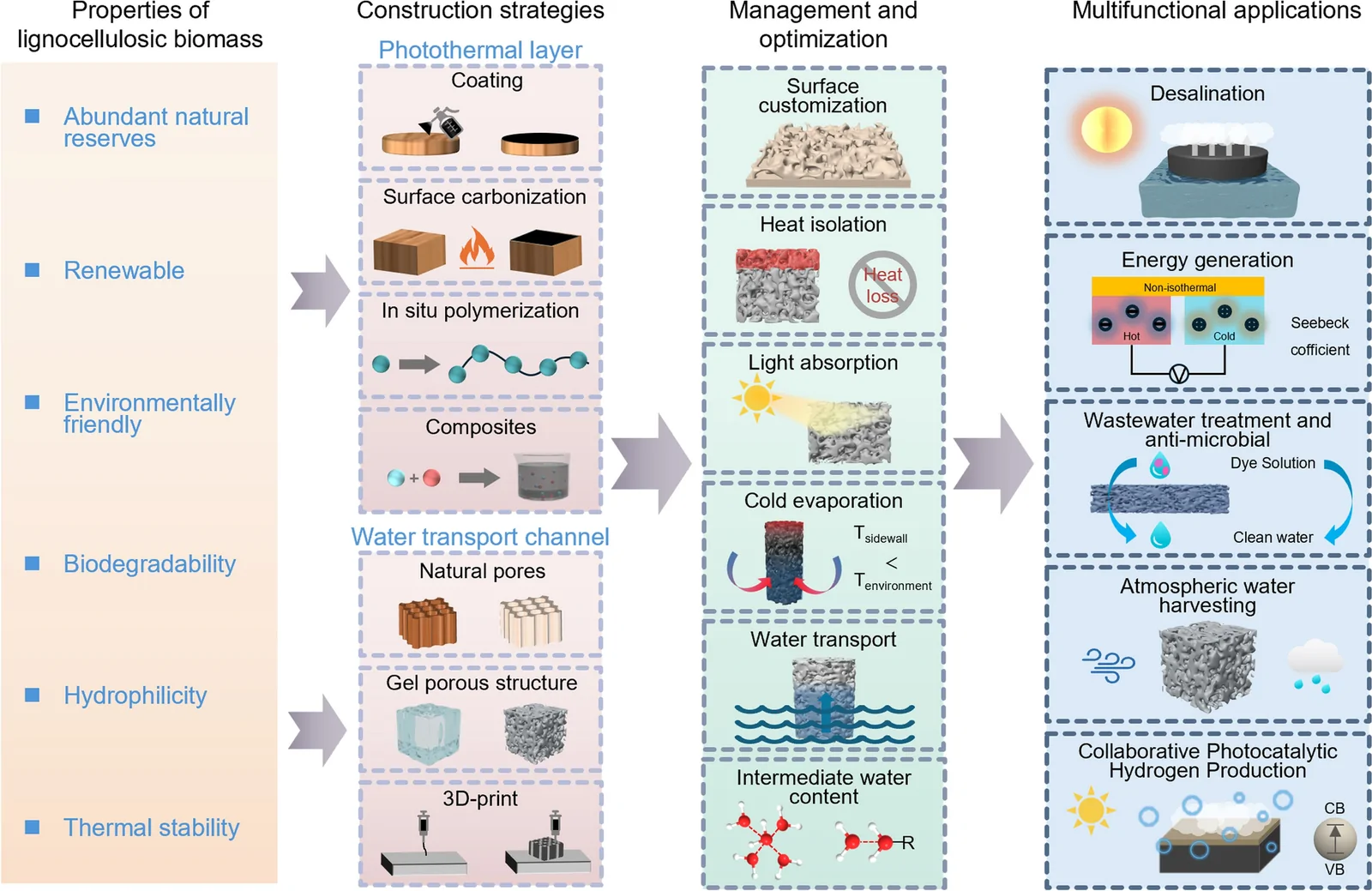
Summary of the properties–construction strategies–management and optimization of lignocellulosic biomass and multifunctional applications

A typical SDIE comprises a photothermal layer and a supporting substrate with water transport channel. When exposed to sunlight, the photothermal layer absorbs light and converts it into thermal energy (heat), driving the evaporation process. The water transport channel allows for continuous upward water flow to the photothermal interface, where thermal energy converts liquid water to vapor phase. The supporting substrate plays a vital role in providing structural integrity, while its thermal conductivity and mechanical strength are essential for the long-term stability and durability of the system. Therefore, the optimal design and integration of the photothermal layer, water transport channel, and supporting substrate are critical to the efficient operation of the interface evaporator. Lignocellulosic biomass materials, due to their abundance and favorable physicochemical properties, hold significant promise for use in SDIEs. Optimizing the structure of these materials enhances their photothermal efficiency and water transport capabilities, thereby improving SDIEs performance. Additionally, their inherent sustainability makes them ideal for SDIEs. Figure 4 provides a comprehensive summary of lignocellulosic biomass in interface evaporators, highlighting key material properties, construction strategies, and optimization methods. It underscores how the selection and design of these materials can optimize both evaporation efficiency and system stability, while also elucidating the advantages and challenges of their practical application. This figure offers critical insights for guiding the future development and sustainable integration of these materials into lignocellulosic biomass-based SDIEs.

### Photothermal Layer Construction

#### Coating

**Fig. 5:**
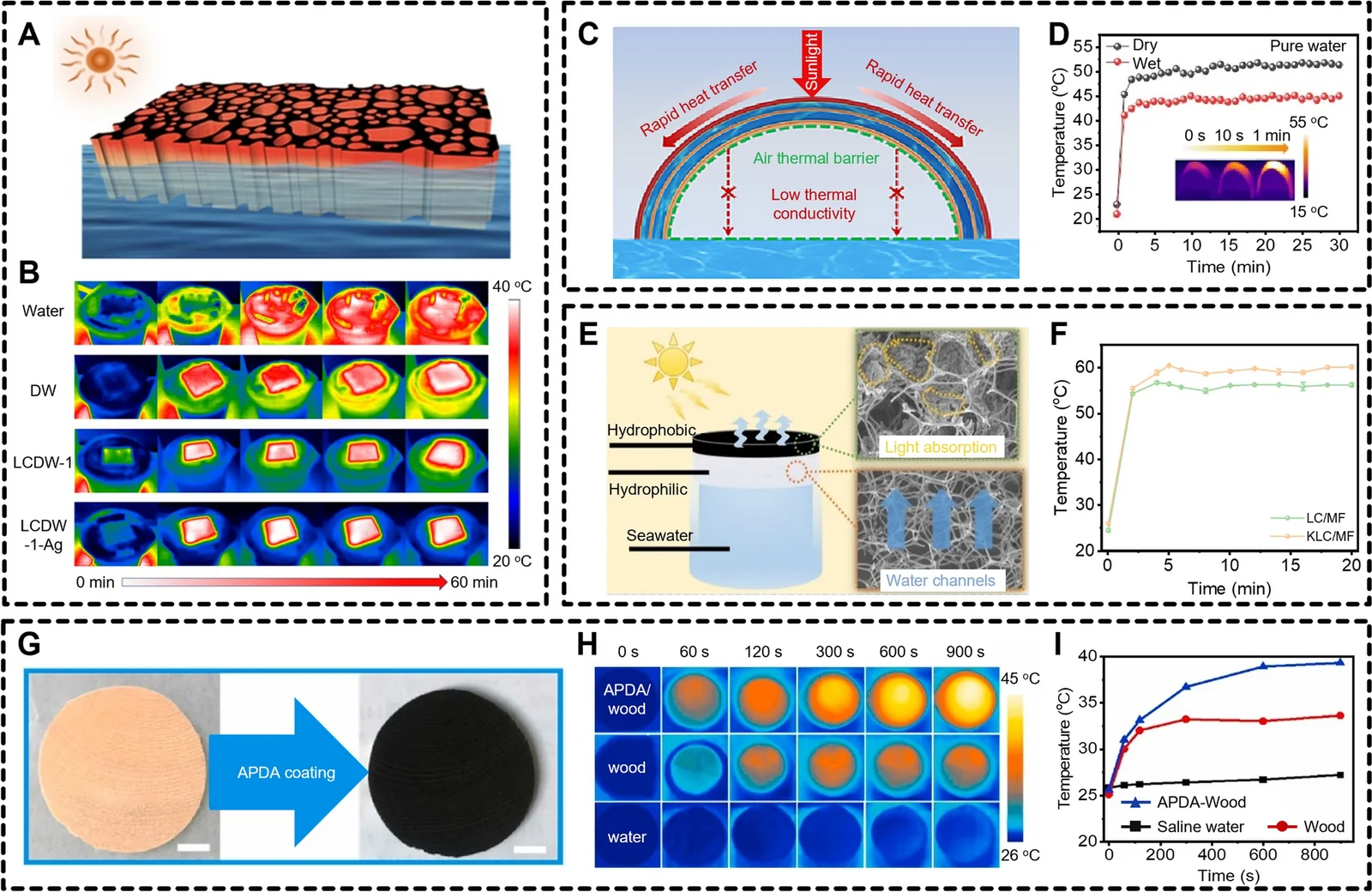
**A** Schematic illustration of the lignin carbon coating on delignification wood (LCDW)-1-Ag SDIE; **B** IR photographs of water, delignified wood (DW), LCDW-1, and LCDW-1-Ag under 1 kW m.^−2^ illumination [34]. **C** Schematic representation of the Janus arch-structured SDIE. **D** Temporal evolution of surface temperature of Janus delignified longitudinal (J-D-L) evaporator operating in wet/dry environments under one sun irradiation. The inset presents infrared thermal images documenting the temperature elevation process under dry conditions [79]. **E** Schematic diagram of the potassium hydroxide-activated lignin-based carbon/melamine foam (KLC/MF) SDIE. **F** Surface temperatures of lignin-based carbon (LC)/MF and KLC/MF at varying illumination durations [80]. **G** Photographs of wood substrate and arginine-doped polydopamine (APDA) wood. **H** Time-resolved infrared thermography of APDA wood, untreated wood, and water under one sun irradiation. **I** The plot displays average surface temperature profiles of APDA-functionalized wood, untreated wood, and brine under standardized solar simulation [81]

Coating technology represents a critical technological foundation for the integration of photothermal conversion interfaces into solar-driven lignocellulosic biomass evaporative systems. These coatings, composed predominantly of high-efficiency photothermal materials, are engineered to harness incident solar radiation with high efficacy and transduce it into localized thermal energy. Implementation is facilitated by versatile coating techniques, including deposition [82], spray coating [83], and dip coating [84], which ensure scalability and compatibility with diverse substrates. Furthermore, such methodologies enable the realization of lightweight and compact evaporative systems by minimizing material usage while maximizing surface-area-to-volume ratios, thereby optimizing overall energy conversion efficiency and operational performance under varied environmental conditions. For instance, Wu et al. [34] reported that Ag nanoparticles (NPs) were anchored onto lignin-derived porous carbon, which was subsequently deposited at the top of the surface of delignified wood for effective light absorption (Fig. 5A). The enhanced solar absorption was attributed to the nanopores within the lignin-derived carbon acting as optical microcavities, promoting multiple scattering and improving the path of incident light [85]. Additionally, the incorporation of Ag nanoparticles created hot spots that enhanced light absorptivity [86]. After 5 min of illumination under 1 sun, the surface temperature of the LCDW-1-Ag composite rose rapidly to 36.5 °C, while the measured temperatures of water, deionized water (DW), and LCDW-1 were recorded at 25.4, 29.2, and 35.8 °C, respectively (Fig. 5B). These results demonstrated that LC-1-Ag exhibited a fast thermal response capability, highlighting its promising potential for efficient solar-driven evaporation application.

In another approach, Chen et al. [79] utilized a commercial black aerosol spray, primarily composed of carbon black, which was used to spray on the surface of delignified longitudinal wood (D-L wood) to enhance light absorption, facilitate solar thermal conversion, and promote steam evaporation, resulting in an evaporation rate of 2.82 kg m^−2^ h^−1^ in pure water (Fig. 5C). The surface temperature of the J-D-L wood can rapidly reach 45 °C within one minute and ultimately stabilize at approximately 51 °C under dry conditions when exposed to AM 1.5 solar radiation (Fig. 5D). Moreover, Li et al. [80] reported the synthesis of activated lignin-based carbon (KLC) through KOH activation, which was subsequently coated onto the upper surface of commercial melamine foam (MF) to fabricate a self-floating Janus KLC/MF evaporator (Fig. 5E). The porous architecture of KLC demonstrated exceptional solar energy harvesting capabilities, achieving 90% broadband absorption across the full solar spectrum (200–2500 nm) and efficient photothermal conversion with equilibrium temperatures reaching 60.4 °C under standard illumination (Fig. 5F). As a result, the Janus KLC/MF evaporator demonstrated a remarkable water evaporation rate of 1.539 kg m⁻^2^ h⁻^1^ under one solar irradiation. Similarly, Zou et al. [81] developed a photothermal-enhanced arginine-doped polydopamine (APDA) (Fig. 5G). The APDA coating exhibits superior optical absorption and photothermal conversion efficiency relative to conventional polydopamine (PDA) coatings, a performance enhancement attributable to donor–acceptor pair formation in its microstructure. The APDA wood composite exhibited rapid thermal response under 1-sun illumination, reaching an average surface temperature of 38 °C within just 5 min and stabilizing at nearly 40 °C (Fig. 5H, I). This rapid temperature rise highlights the exceptional light absorption capacity and effective photothermal performance by coating technology.

#### Surface Carbonization

**Fig. 6:**
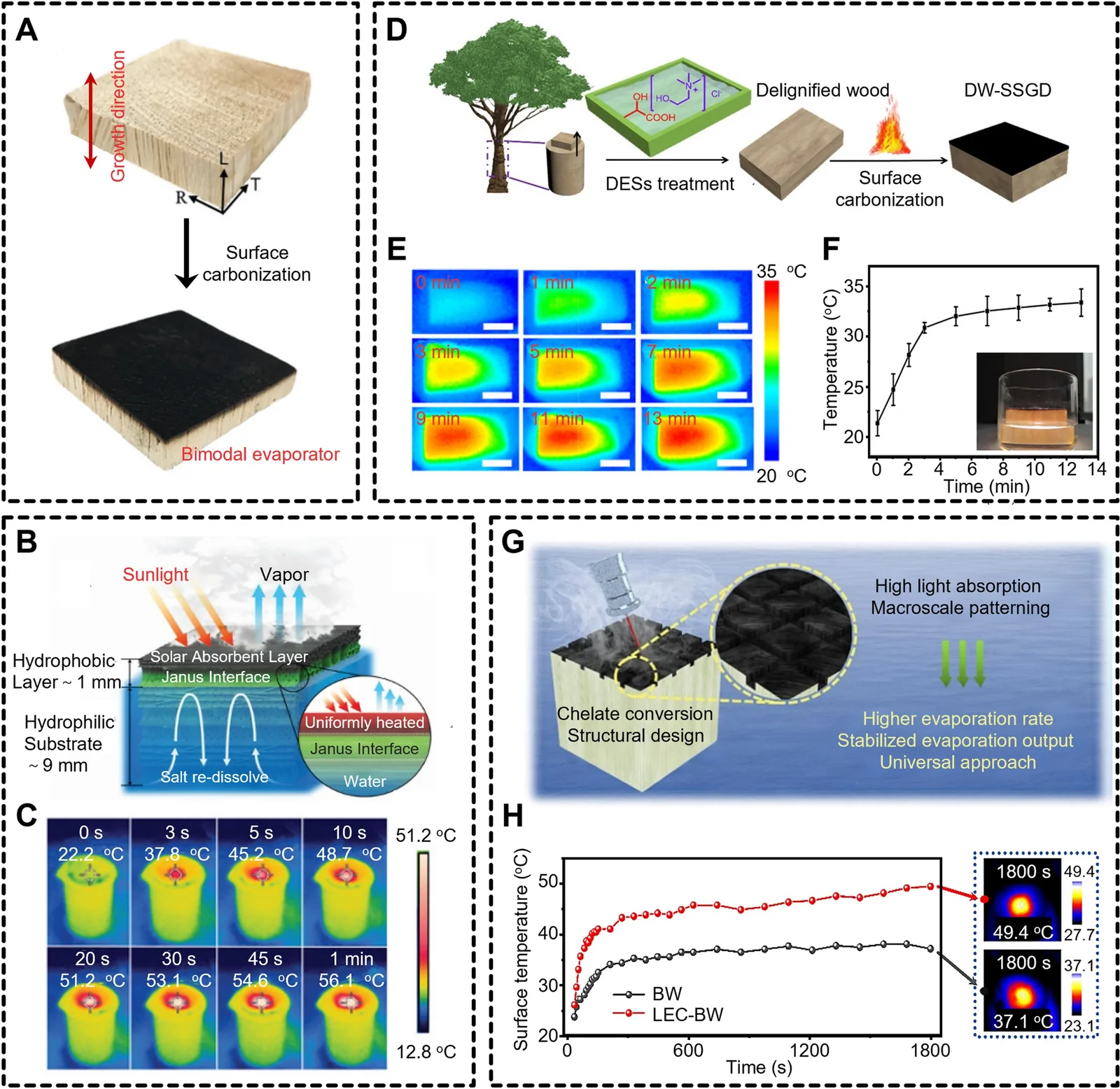
**A** Optical images comparing untreated balsa wood with the bimodal-structured, salt-rejecting SDIE [87]. **B** Schematic of a Janus wood SDIE. **C** Thermography captures the desalination module's surface temperature evolution under concentrated solar flux (3-sun illuminations) over a 60-s duration [88]. **D** Schematic illustration of the delignified wood-based solar steam generation devices (DW-SSGD) preparation. Thermal profiling of the DW-SSGD interface via **E** synchronized infrared thermography and **F** temperature measurements under standardized solar simulation [35]. **G** Schematic of the proposed concept of chelate conversion and structural design by laser engraving. **H** Surface temperature profiles of laser engraving balsa wood with chelation treatment (LEC-BW) and balsa wood (BW) specimens under 1-sun illumination with corresponding infrared thermograms recorded at the 1800s [89]

Surface carbonization is a foundational strategy for the construction of photothermal layer in lignocellulosic biomass-based SDIEs, as it generates a graphitic photothermal conversion layer that markedly improves solar absorption and thermal localization. This carbonization process, which transforms the wood surface into a porous, light-absorbing matrix, serves as a scalable and energy-efficient route for material functionalization. Widely adopted techniques, such as heated plate annealing [90], controlled flame carbonization [91], and precision laser irradiation [92], enable tunable surface morphology and chemical composition, thereby tailoring light-to-heat conversion kinetics. Furthermore, the inherent structural hierarchy of carbonized wood synergizes with these methods to enhance interfacial evaporation rates while maintaining mechanical robustness, offering a sustainable pathway for high-performance biomass-derived evaporators. For example, He et al. [87] introduced a pressure-assisted carbonization method to create a bimodal porous wood film with a carbonized surface by applying pressure to wooden blocks on a hot plate at 500 °C (Fig. 6A). This carbonized film functions as a salt-accumulation-free SDIE, enabling continuous, stable, and efficient desalination of high-salinity water. The stabilized temperature on the top surface of the SDIE increases with light intensity. Similarly, Chen et al. [88] adopted a comparable carbonization technique, in which the wood was tightly pressed on a 500 °C hot plate to develop a sustainable Janus wood SDIE featuring a carbonized surface (Fig. 6B). This evaporator achieved an evaporation efficiency of 82.0% for a 20% NaCl solution under 1-sun illumination. Under identical irradiation conditions, the Janus wood achieved a 56.1 °C surface temperature within one minute—an 8.9 °C greater temperature increase than natural wood (47.2 °C), with both materials starting from comparable initial temperatures (Fig. 6C).

In a different approach, Chen et al. [35] applied a deep eutectic solvent (DES) to extract lignin from bulk wood under mild conditions, followed by treatment with a scanning flame to create a solar-to-thermal layer (Fig. 6D). Under simulated solar radiation, the surface temperature of the delignified wood with scanning flame treatment rapidly increased from approximately 28 to 60 °C over 13 min (Fig. 6E). Under 1 standard sun irradiation, the surface temperature of the DW-SSGD rose swiftly from about 22 to 33 °C within 4 min (Fig. 6F). Furthermore, Pang et al. [89] proposed a laser engraving-driven integrated approach for carbonizing and modifying the wood surface, achieving simultaneous conversion into carbon and metal oxides while constructing surface architectures (Fig. 6G). The laser engraving technique not only facilitates carbonization but also creates a patterned surface structure that enhances photothermal conversion, water transport, and salt inhibition. The surface temperature of the LEC-BW can be rapidly increased from room temperature to approximately 39.3 °C within 1 min, significantly outperforming untreated wood that reaches only 29.5 °C under the same conditions (Fig. 6H). As a result, it demonstrates a significantly enhanced and stabilized output, achieving evaporation rates of approximately 1.72 kg m^–2^ h^–1^, which exceeds that of pristine wood by about 100%.

#### In Situ* Polymerization*

In situ polymerization has emerged as a pivotal methodology for augmenting the photothermal efficiency of solar evaporators through the synergistic integration of functional light-absorbing polymers, such as polypyrrole (PPy), polydopamine (PDA), and polyaniline (PANi). This technique facilitates covalent bonding and uniform dispersion of photothermal phases within polymeric matrices, thereby ensuring robust interfacial adhesion while simultaneously optimizing broad band solar absorption and heat generation—critical parameters for high-yield solar desalination. By enabling atomically controlled deposition of conformal polymer coatings, in situ polymerization enhances thermal conductivity, operational stability, and anti-fouling resistance against salt crystallization, addressing persistent challenges in long-term evaporator durability. Moreover, the scalability and cost-effectiveness of this approach position it as a transformative pathway for engineering next-generation SDIEs, with implications for advancing sustainable water purification technologies in resource-limited settings.

**Fig. 7:**
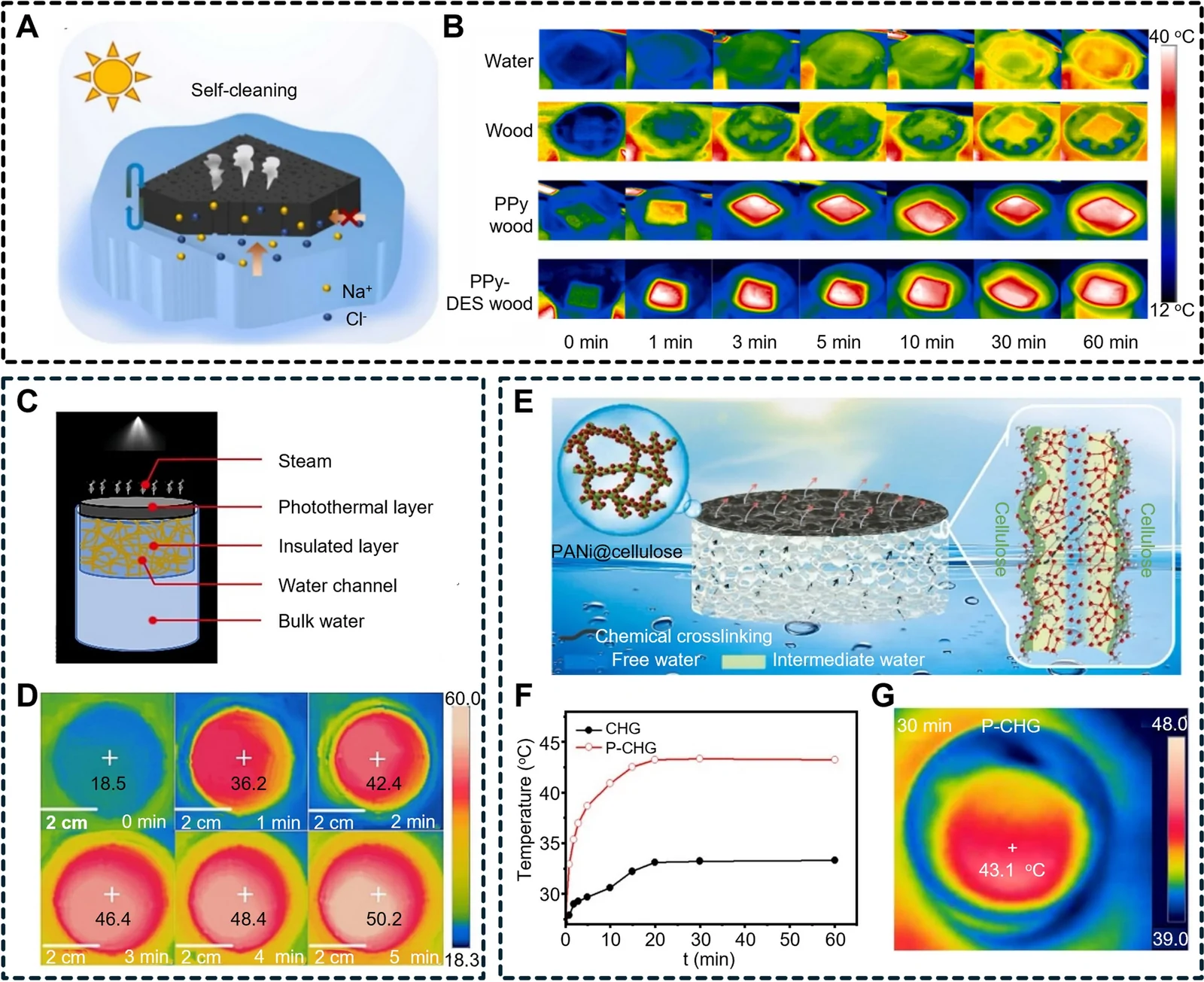
**A** Schematic illustration of polypyrrole-deep eutectic solvent (PPy-DES) wood with autonomous surface-cleaning capability. **B** Infrared thermal mapping of bulk water, untreated wood, PPy wood, and PPy-DES wood surfaces [93]. **C** Schematic illustration of polydopamine-functionalized lignin containing pulp (LPNR@PDA) foam evaporator. **D** Mass changes of seawater, lignin containing pulp natural rubber (LPNR) foam, and LPNR@PDA foam evaporator under standard solar flux [94]. **E** Schematic illustration of polyaniline (PANI) @cellulose evaporator. **F** Surface thermal evolution under 1-sun irradiation, with **G** corresponding infrared thermography revealing spatial temperature gradients [36]

For example, Shen et al. [93] demonstrated the utility of in situ polymerization by functionalizing wood substrates with a deep eutectic solvent (DES), which exposed abundant cellulose hydroxyl groups via selective lignin removal. This surface modification facilitated hydrogen-bond-mediated deposition of conformal PPy coatings, yielding a low-cost, high-efficiency solar interfacial evaporator (Fig. 7A). The PPy-coated wood evaporator achieved a rapid thermal equilibrium, attaining a surface temperature of 37.8 °C within 10 min. The DES-functionalized PPy wood (37.8 °C) evaporator achieved a higher equilibrium temperature than unmodified PPy wood (34.8 °C) (Fig. 7B). Similarly, PDA is a prominent photothermal material renowned for its broadband absorption and biocompatibility [95]. Zhang et al. [94] engineered a lightweight, porous LPNR@PDA foam evaporator via in situ polymerization (Fig. 7C). The LPNR@PDA foam evaporator exhibited surface temperatures of 28.7, 30.8, 35.1, and 43.3 °C under 1-, 2-, 3-, and 5-sun irradiance, respectively, within 5 min (Fig. 7D), demonstrating superior photothermal conversion kinetics across varying solar fluxes. These comparative studies highlight the versatility of in situ polymerization in tailoring evaporator architectures for optimized energy harvesting, stability, and scalability in solar-driven desalination. PPy and PDA exhibit significant promise for the preparation of SDIEs, due to their intrinsic capacity for broadband solar absorption and efficient light-to-heat conversion [96]. Similarly, PANi has emerged as a robust photothermal material, leveraging its *π*-conjugated backbone to form stable interactions with cellulose matrices [97]. Shu et al. [36] demonstrated this synergy by fabricating a cellulose hydrogel evaporator via in situ PANi polymerization on a cellulose network (Fig. 7E). The PANi-coated hydrogel achieved rapid thermal equilibration, with surface temperatures rising to 43.1 °C within 5 min under solar irradiation and stabilizing after 30 min (Fig. 7F, G). This accelerated thermal response underscores exceptional photothermal localization of PANi, attributed to enhanced photon capture and minimized thermal dissipation at the polymer–cellulose interface.

#### Synergistic Composites

Conventional interfacial evaporators employing singular photothermal materials face intrinsic limitations, such as narrow absorption spectra, suboptimal conversion efficiencies, and environmental instability, impeding their practical applications. The integration of heterostructured composites, which synergize distinct photothermal mechanisms, offers a transformative pathway to overcome these constraints. This strategy of synergistic composite design has emerged as a pivotal approach for constructing advanced photothermal layers, enabling enhanced performance through the combination of multiple functional materials.

**Fig. 8:**
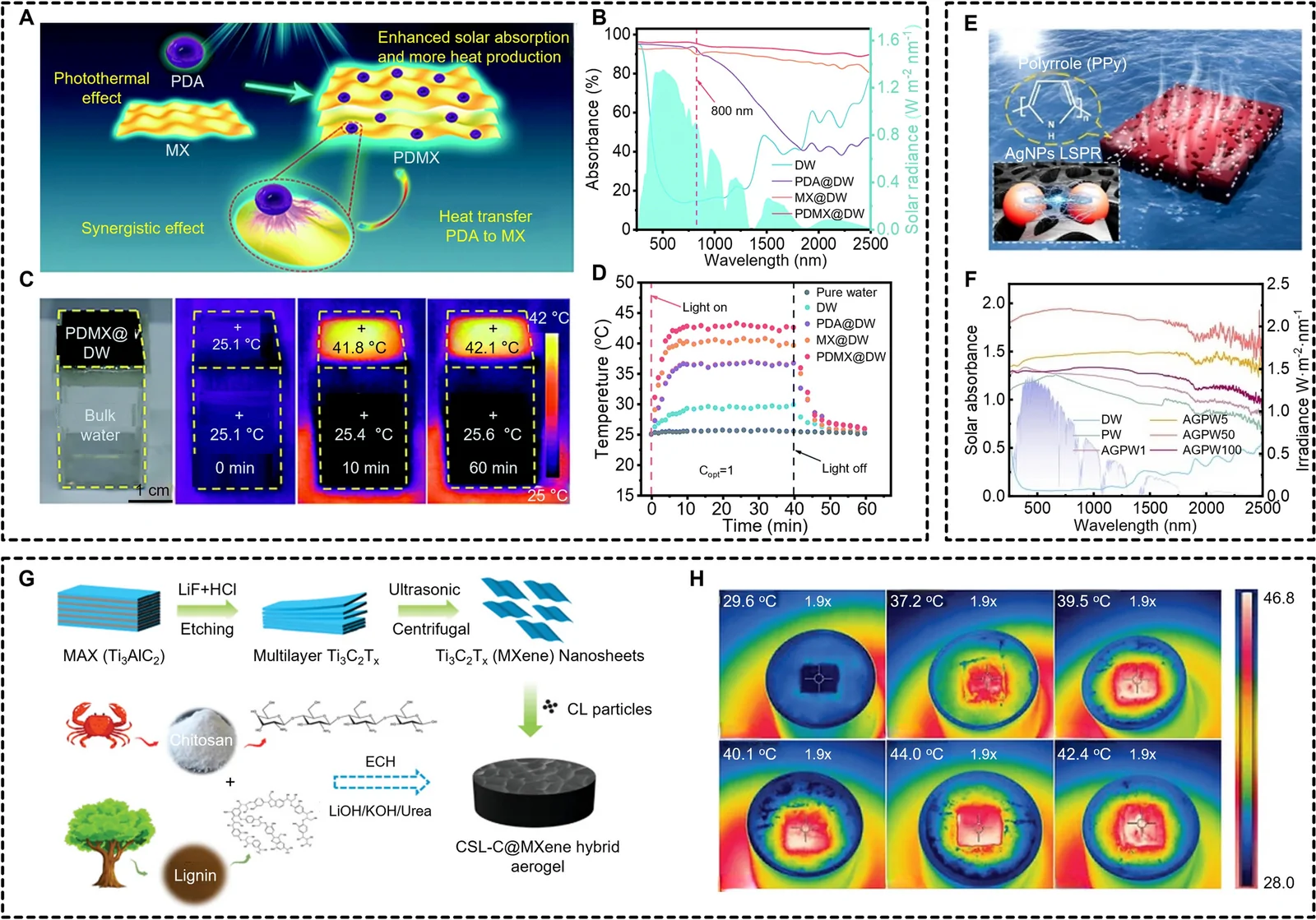
**A** Schematics illustrating the cooperative photothermal coupling between PDA and MXene (MX) nanostructures under solar irradiation. **B** Absorbance profiles of diverse samples. **C** Visual documentation of the evaporation system and IR thermal images at different time intervals (0, 10, 60 min) under 1 sun. **D** Temperature of various samples in pure water as a function of time [9]. **E** Diagram of the Ag/PPy wooden SDIE. **F** Comparative UV–Vis–NIR spectroscopy results for delignified wood (DW) and its PPy (PW) and Ag/PPy (AgPW) modified versions [37]. **G** Schematic representation of fabrication of CSL-C@MXene (chitosan/lignin (CSL) aerogel as the skeleton, loaded with light-harvesting carbonized lignin (CL) particles and Ti3C2TX (MXene) nanosheets. **H** UV–Vis–NIR absorption spectrum for CSL, CSL@MXene-20 mg, and CSL-C@MXene [98]

For instance, Chen et al. [9] engineered a hierarchical photothermal interface by co-depositing PDA and Ti_3_C_2_T_x_ MX on DW via mussel-inspired polymerization (Fig. 8A). The unique intercalated PDA-MX structure with expanded layer spacing resulted in effective solar capture ability (Fig. 8B) and enhanced its multi-scattering effects. Under 1-sun irradiation, the PDMX@DW evaporator attained a surface temperature of 42.1 °C within 5 min, which is 17 °C higher than ambient water temperature, while maintaining bulk water at 25.4 °C, demonstrating exceptional thermal localization (Fig. 8C, D). The synergistic interaction between PDA and MXene exemplifies the effectiveness of composite design in optimizing photothermal performance. Complementing this, Lu et al. [37] developed an Ag/PPy-decorated wood evaporator (Fig. 8E), where plasmonic Ag nanoparticles and conjugated PPy coatings synergistically enhanced broadband absorption (Fig. 8F). The hybrid system leveraged nonradiative relaxation of vibrational modes [99] of PPy and the localized surface plasmon resonance effect [100] and plasmonic heating of Ag [101], achieving an evaporation rate of 2.04 kg m⁻^2^ h⁻^1^, higher than single-component counterparts. This design highlights the potential of combining plasmonic and conjugated polymer materials to create efficient photothermal layers.

While, Chen et al. [98] synthesized a chitosan-lignin/MXene aerogel (CSLC@MXene) with dual photothermal pathways (Fig. 8G). Carbonized lignin particles (CL) provided molecular vibration-driven heating, while MXene nanosheets induced plasmonic resonance, collectively elevating the surface temperature to 44.0 °C under 1 sun (Fig. 8H). This interfacial synergy reduced heat loss compared to unitary systems, underscoring the efficacy of composite design in balancing efficiency and durability. The integration of dual photothermal mechanisms in this composite material demonstrates the versatility of synergistic design in photothermal layer construction. These advances highlight the pivotal role of multi-mechanistic photothermal synergistic composite in advancing solar desalination technologies, bridging the gap between laboratory innovation and scalable, environmentally resilient systems. The strategy of synergistic composite design not only addresses the limitations of single-material systems but also provides a robust framework for developing high-performance photothermal layers tailored for practical applications.

The establishment of a high-performance photothermal layer, while necessary, is an insufficient condition for achieving high-efficiency SDIEs. A further critical determinant is the establishment of a mechanism for the continuous and stable transport of water to the evaporation interface, ensuring its effective coupling with the localized thermal energy. Within this framework, the photothermal layer functions to convert solar radiation into thermal energy, whereas the water transport channel is tasked with supplying a constant flux of liquid water.

### Water Transport Channel Construction

#### Natural Pores in Wood

**Fig. 9:**
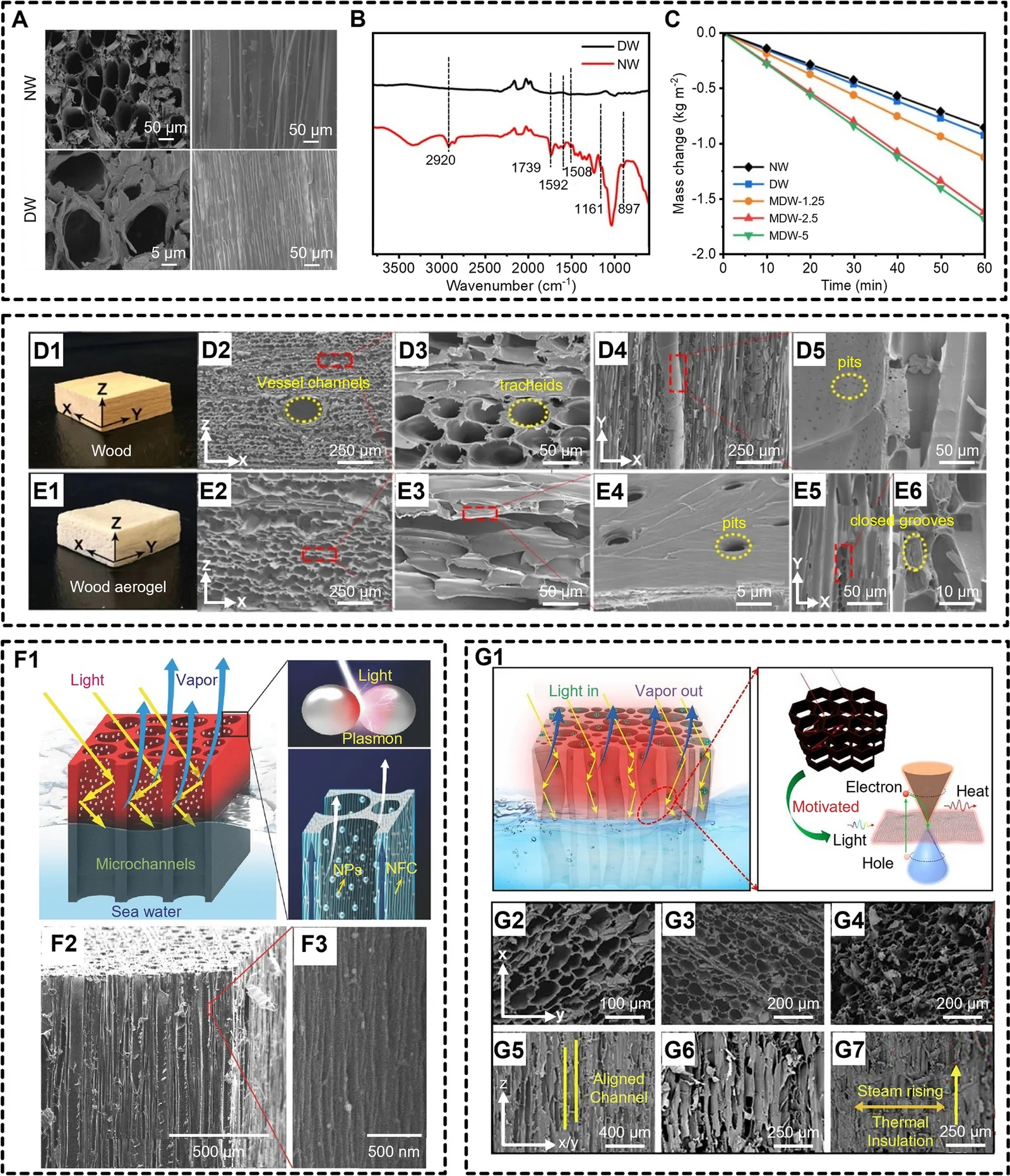
**A** Top-view and cross-sectional SEM images and **B** infrared absorption spectrum of delignified wood (DW) and nature wood (NW) samples. **C** Mass change during 1-sun exposure for NW, DW, MXene @DW (MDW)-1.25, MDW-2.5, and MDW-5 samples [102]. **D1** Photographic depiction of pristine balsa wood. **D2**, **D3** SEM characterization demonstrates balsa wood's porous morphology, exhibiting orderly arranged microchannels with distinct vascular features: large vessels (200–300 μm) and narrower tracheids (50–80 μm). **D4**, **D5** SEM characterization of balsa wood showing frequent pit features in its cell wall morphology. **E1** Image of the aerogel material derived from wood. **E2**, **E3** Electron microscopy images display the hierarchical organization of wood-derived aerogel, showing sequential arch-like lamellae. **E4** SEM micrographs displaying porous pit features on the walls of aerogel. **E5**, **E6** SEM images reveal sealed groove structures within the wood-based aerogel [103]. **F1** Schematic illustration of plasmonic wood. **F2** discloses the well-organized mesoporous system in plasmonic wood, comprising open, oriented microchannel structures and **F3** SEM image showing the microchannel walls formed by parallel-arranged cellulose nanofibrils [1]. **G1** Schematic of lignin-derived carbon quantum dots on delignified wood (LCQDs-DW) demonstrating its photothermal evaporation under sunlight, with the carbon hexatomic ring structure enhancing light absorption and conversion. **G2**–**G4** Cross-sectional SEM images of wood, DW, and LCQDs-DW, respectively. **G5**–**G7** SEM micrographs of longitudinal sections from wood, DW, and LCQDs-DW, respectively [104]

The hierarchical pore structures in natural wood enable exceptional water storage capacity (100–170%) and unidirectional transport, driven by capillary forces within aligned microchannels and pit-mediated lateral pathways [54, 105–108]. Lower-density variants exhibit higher porosity, enhancing hydraulic conductivity while maintaining buoyancy, a critical feature for floating evaporators [109]. In addition, delignification further optimizes this innate structure. He et al. [102] demonstrated that lignin removal preserves vertical microchannel orientation of wood (Fig. 9A) while eliminating C–H (2920 cm⁻^1^) and C=O (1739 cm⁻^1^) vibrational modes, as confirmed by FT-IR (Fig. 9B). The resulting DW substrate achieved a 23% increase in evaporation efficiency compared to NW by reducing light-blocking aromatic moieties (Fig. 9C).

Structural engineering extends beyond lignin extraction. Zhang et al. [103] developed a flexible and mildew-resistant aerogel derived from natural balsa wood to serve as an efficient and stable substrate for solar desalination applications. The balsa wood exhibits a three-dimensional interconnected porous network, consisting primarily of large vessel channels (200–300 μm) and narrow tracheids (50–80 μm). These well-aligned microchannels facilitate rapid water transport along the direction of tree growth. Additionally, the vessel channel cell walls contain numerous micropores (1–3 μm), which facilitate lateral water movement (Fig. 9D). In contrast to natural wood, the microstructure transitions of wood-derived aerogels from orderly elliptical cavities to an arch-layered lamellar arrangement. These lamellar layers retain open micropores on their surfaces (Fig. 9E), which act as channels for brine transport and pathways for salt diffusion. Functionalization via photothermal material integration unlocks further potential. Zhu et al. [1] embedded plasmonic nanoparticles within mesoporous framework of wood, creating a “plasmonic wood” evaporator with near-unity solar absorption (99%, 200–2500 nm) and low-tortuosity water pathways (Fig. 9F). Similarly, Chao et al. [104] coupled delignified wood with LCQDs, achieving a 44.0 °C surface temperature under 1 sun via synergistic light trapping and steam-conductive channels (80–100 µm, Fig. 9G). These advances underscore the wood versatility as a structurally and functionally tunable water transport channel for high-efficiency solar desalination.

#### Gel Porous Structure

In addition to the utilization of the wood innate porosity, hydrogel offers distinct advantages for SDIEs, such as tunable hydration states, programmable pore architectures, and inherent hydrophilicity [43]. The high crystallinity of cellulose contributes to remarkable mechanical stability, while its hydroxyl-rich surface facilitates direct integration of photothermal nanomaterials without complex functionalization [1, 110]. Combined with low thermal conductivity, these properties minimize parasitic heat loss, enabling efficient solar-to-thermal energy confinement [50, 111]. Advanced ice-templating techniques, such as directional freezing, further refine hydrogel structures to mimic aligned microchannels of wood for rapid water transport [112].

**Fig. 10:**
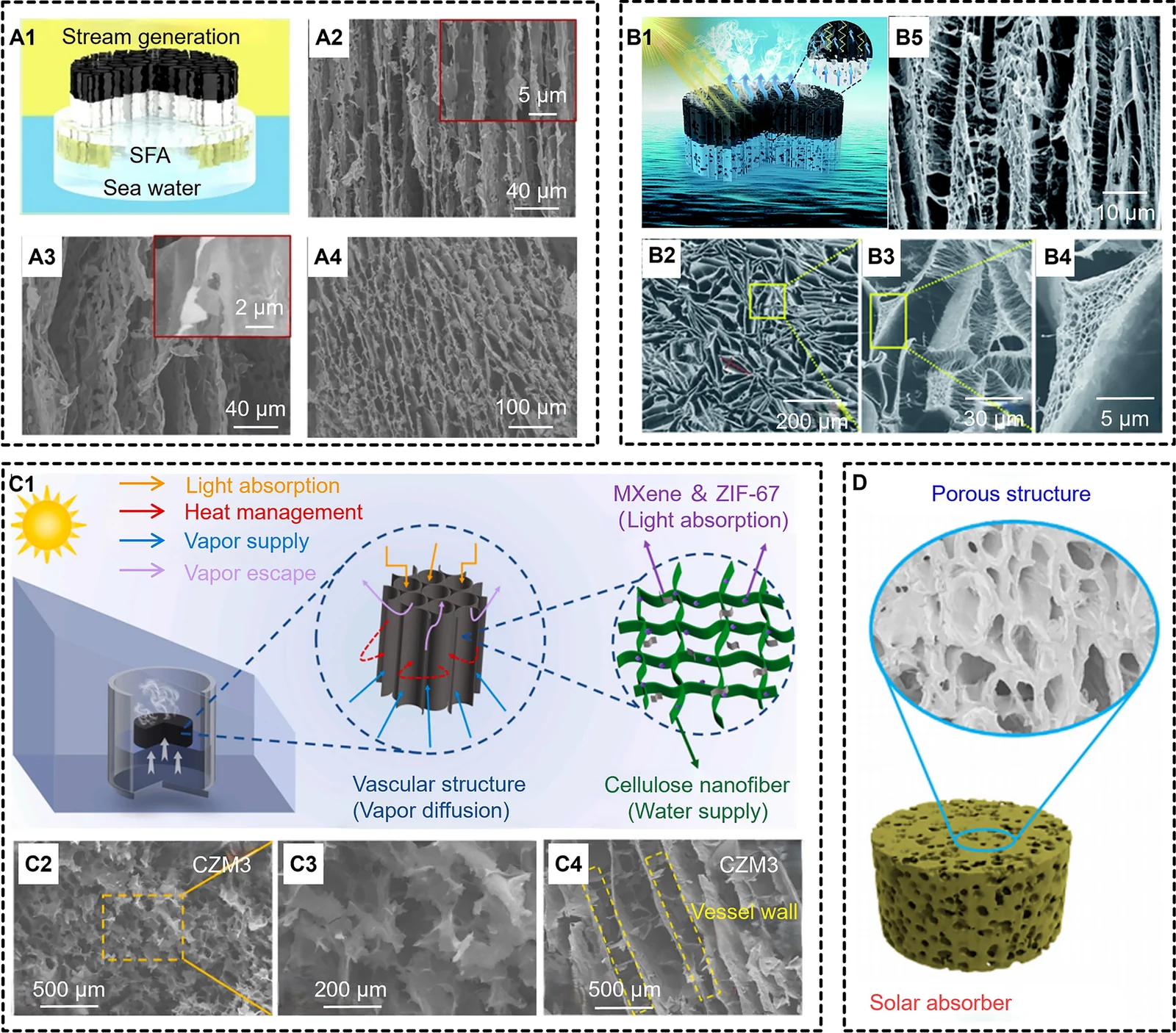
**A1** Schematic illustration of the double-layered regenerated cellulose (RC)/regenerated cellulose (CS) aerogel (DLRCA). Vertical sectional SEM images of **A2** RC/CS aerogel frozen in liquid nitrogen (RCA-LN), **A3** regenerated cellulose aerogel (RCA). Cross-sectional SEM images of **A4** RCA [113]. **B1** Schematic illustration of Janus cellulose nanofibril (CNF)/MXene composite (JCM) aerogels. **B2**–**B4** SEM images of cross-section across varying magnification levels **B5** SEM images of lateral surface [45]. **C1** Diagrammatic overview of the experimental device for SDIE. Top-view SEM image of **C2**, **C3** cellulose nanofiber with ZIF-67 and MXene (CZM3) aerogels. **C4** Side-view SEM images [114]. **D** Scheme of polyvinyl alcohol (PVA)/ sodium lignosulfonate (SLS)-CNT hydrogel [77]

Zhou et al. [113] engineered a bilayer cellulose aerogel from waste cotton fabrics, employing chitosan-assisted ice templating to create hierarchical pores (Fig. 10A). Precise thermal control yielded vertically aligned channels (40–60 µm) in the RCA, while liquid nitrogen quenching produced finer mesopores (5–10 µm), enhancing capillary-driven water flux. Han et al. [45] extended this approach by fabricating Janus aerogels via unidirectional freeze-drying of CNFs and Ti3C2Tx MXenes (Fig. 10B). The MXene-CNF crosslinked network prevented nanosheet stacking, yielding spindle-shaped macropores (86 × 28 µm^2^) and vertically aligned microchannels that reduced thermal conductivity by 32% while maintaining 89% solar absorption. Zhou et al. [114] further optimized vascular-like structures by integrating ZIF-67 and MXene into CNFs (Fig. 10C). Hydrogen bonding of MXene strengthened the CNF matrix, forming an interlocked network with dual-scale pores (150–200 µm vessels; 50–100 µm veins) that achieved an evaporation rate of 1.85 kg m⁻^2^ h⁻^1^ under 1 sun. While pure lignin lacks structural coherence for standalone substrates, its sulfonated derivatives address limitations of cellulose in hypersaline environments. Hao et al. [77] incorporated SLS into hydrogels, leveraging sulfonate and hydroxyl groups to enhance hydrophilicity and intermediate water content (Fig. 10D). The SLS-cellulose composite reduced vaporization enthalpy by 18% and achieved a record evaporation rate of 2.09 kg m⁻^2^ h⁻^1^, demonstrating lignin untapped potential in modulating water state dynamics.

#### 3D Print

**Fig. 11:**
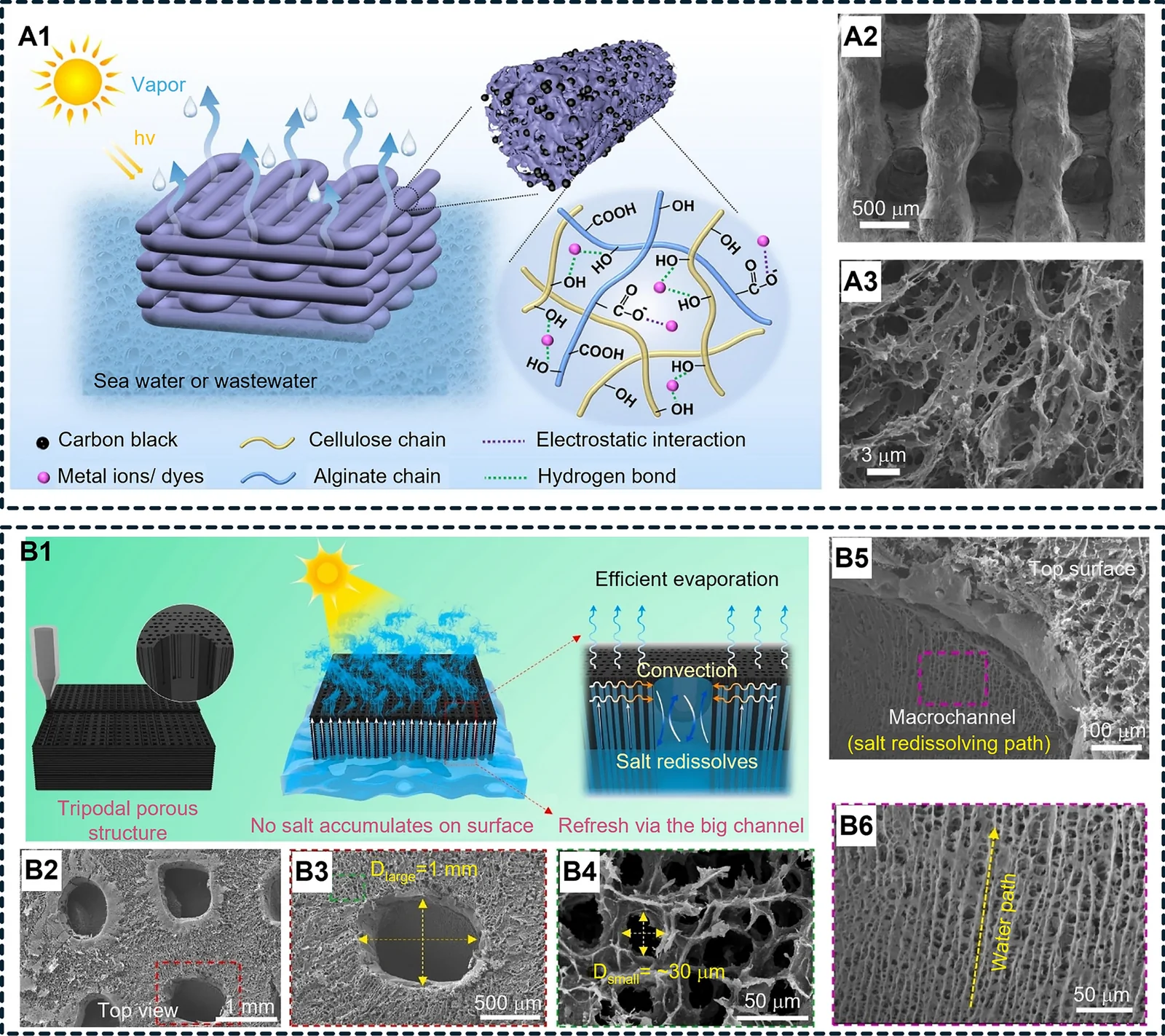
**A1** Schematic illustration of SDIE based on 3D-printed cellulose/alginate/carbon black hydrogel (CACH). SEM images of the surface **A2**, **A3** of the evaporator [115]. **B1** 3D-printed tripodal evaporator with salt-rejecting macroporous architecture. **B2**–**B4** Surface morphology characterization by SEM at varying magnifications (top-view) **B5**, **B6** SEM images showing internal pore structure [44]

3D printing has revolutionized the design of cellulose-based solar evaporators by enabling precise control over pore architecture, spanning macroscale water channels to submicron voids, to optimize hydraulic and photothermal performance. For example, Yuan et al. [115] utilized 3D printing method to fabricate a carbon black-embedded cellulose hydrogel (CACH) with triphasic porosity (Fig. 11A). The structure integrates 3D-printed macropores (0.50 ± 0.05 mm) for vapor escape, hydrophilic mesopores (5–10 µm) for capillary pumping, and nanoscale cellulose fibrils for interfacial water confinement, achieving an evaporation rate of 1.33 kg m⁻^2^ h⁻^1^ under 1 sun. Chen et al. [44] mimicked multiscale fluidics of wood via a tripodal cellulose composite evaporator (Fig. 11B). The 3D-printed tripodal porous evaporator: (1) 1 mm macrochannels for salt redissolution, (2) 30 µm micropores enabling convection-driven flow, and (3) 1 µm submicron pores sustaining capillary ascent in 15 wt% NaCl brine. This biomimetic design maintained 75% evaporation efficiency over 100 h by synergizing salt rejection and rapid water replenishment.

## Management and Optimization

### Heat Management

#### Surface Customization

**Fig. 12:**
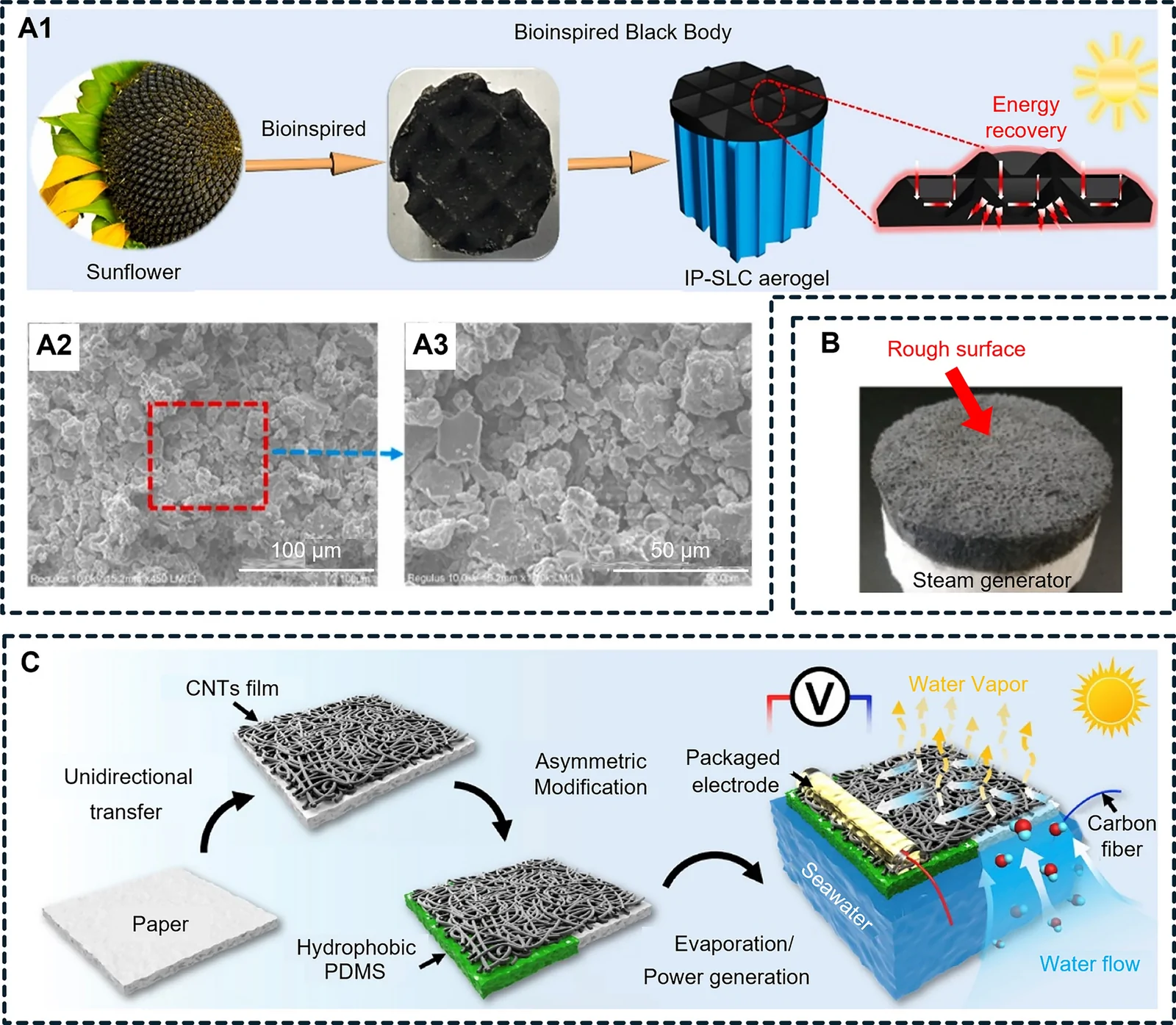
**A1** Schematic illustration of the surface of IP-SLC (inspired by the seedless sunflower, narrow the bandgap of gallium and indium alloy using stearic acid/lignin-cellulose nanocrystals) aerogel inspired by sunflower. **A2** Overhead SEM micrograph. **A3** High-magnification SEM micrograph (top-view) [38]. **B** Digital photograph of all cellulose-based evaporator with a rough surface [7]. **C** Diagram illustrating the manufacturing process of carbon nanotubes/polydimethylsiloxane/paper composite (CPPH) for a dual-functional evaporator with simultaneous steam and power production [6]

Surface topography engineering has emerged as a critical strategy for enhancing light absorption in solar evaporators by leveraging multi-scale scattering and anti-reflective architectures [116–118]. For example, Wei et al. [38] pioneered a bioinspired approach, engineering a lignin-cellulose nanocrystal (CNC) aerogel with inverted pyramid microstructures mimicking the light-trapping morphology of seedless sunflowers (Fig. 12A). The topological synergy between eutectic gallium–indium (EGaIn) and lignin reduced reflectivity, achieving a record solar evaporation efficiency of 94% under 1 sun. Li et al. [7] developed an all-cellulose-based interfacial steam generator, with template-assisted modulation by utilizing abrasive paper to create a rough textured photothermal layer surface (Fig. 12B), which achieving an evaporation rate of 1.82 kg m⁻^2^ h⁻^1^ under ambient conditions.

Surface customization not only improves light absorption but also imparts materials with specialized functionalities. Xiao et al. [6] present an asymmetric CNTs-cellulose paper-PDMS hybrid that simultaneously achieves water evaporation and power generation (Fig. 12C). To create a controllable water flow pathway, the cellulose paper was asymmetrically modified with hydrophobic PDMS at specific locations on one side. This design generated a centimeter-scale water channel that drove directional flow, enabling consistent power output regardless of solar conditions.

#### Heat Isolation

**Fig. 13:**
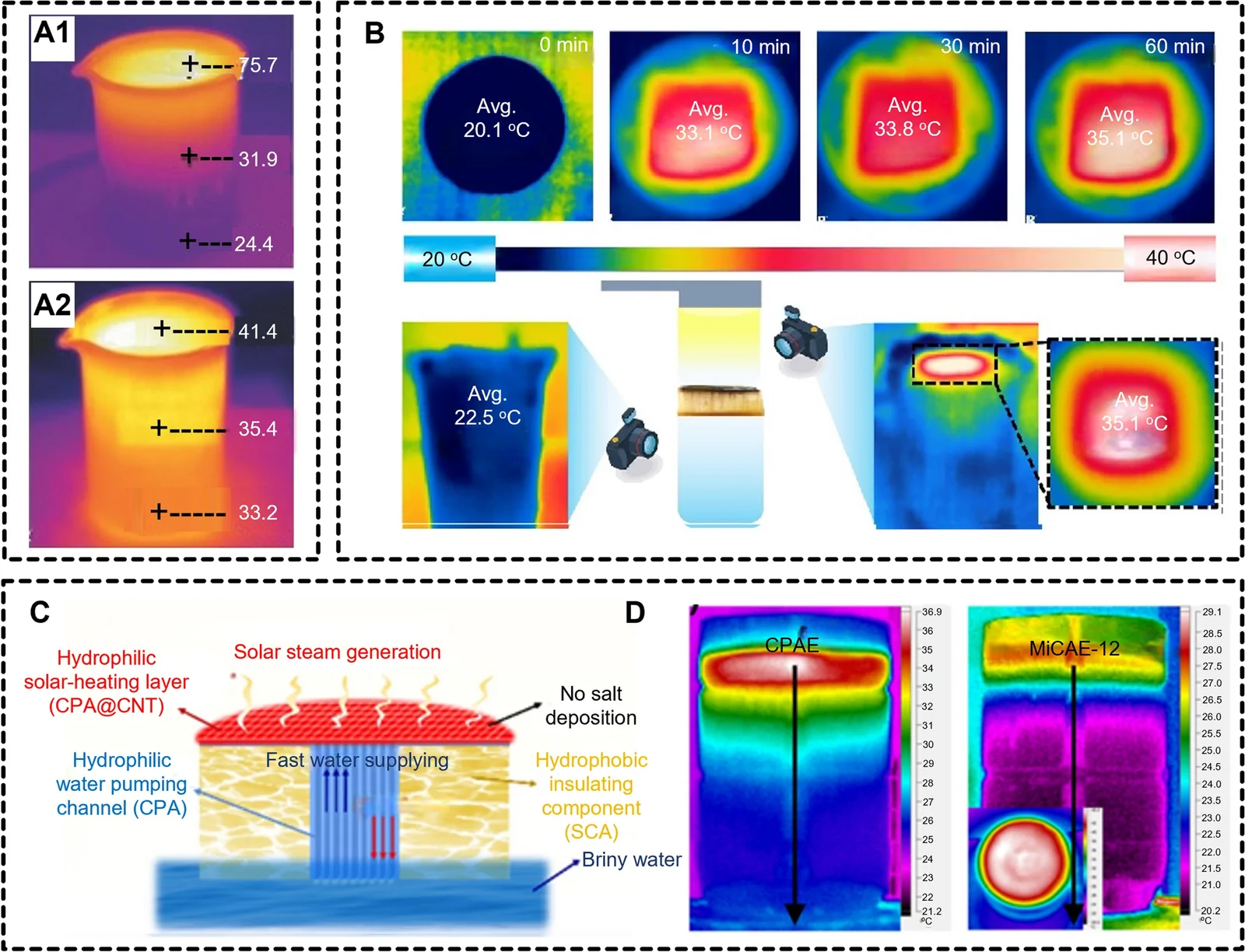
IR images of a water beaker **A1** with or **A2** without the TW-2PDA (polydopamine-coated wood) sample, taken after 10 min of 3.5-sun simulated sunlight irradiation. Temperatures at various positions in the beaker are labeled [120]. **B** Infrared thermal images (top and side views) of photocatalyst-integrated porous carbonized wood-based hydrogels (Hy-P-CW) acquired under one sun irradiation [121]. **C** Schematic diagram illustrating the architecture of the monolithically integrated cellulose aerogel-based evaporator (MiCAE) employed in seawater desalination. **D** IR images of cellulose − PVA aerogel evaporator (CPAE) and MiCAE-12 exposed to simulated solar light at 5-sun intensity in an aqueous environment for 10 min [39]

The anisotropic thermal conductivity of natural wood, rooted in its hierarchical microchannel architecture, enables exceptional thermal insulation critical for interfacial evaporation systems [119]. For example, Wu et al. [120] harnessed this property by coating wood with PDA, creating a photothermal interface that localized surface temperatures to 75.7 °C while maintaining bulk water at ambient levels**.** This stark thermal gradient—absent in control systems—highlights the capacity of wood to confine heat at the evaporation front (Fig. 13A). Fan et al. [121] further advanced this concept by embedding photocatalytic and photothermal agents within delignified wood, achieving rapid surface heating (35.1 °C) without thermal leakage to underlying layers (Fig. 13B), a testament to intrinsic insulating efficiency of wood. Expanding beyond natural materials, synthetic gels replicate and enhance these insulating traits through engineered porosity [122]. Liu et al. [39] developed a robust, floatable MiCAE mimicking fungal and woody structures (Fig. 13C). By synergizing hydrophilic cellulose-PVA, hydrophobic silylated cellulose, and carbon nanotube coatings, the MiCAE minimized radial heat dissipation, maintaining sub-ambient bulk temperatures even under 5-sun irradiation (Fig. 13D). This biomimetic approach underscores the universality of hierarchical structuring in achieving thermal localization, bridging natural and engineered systems for sustainable solar-driven applications.

#### Cold Evaporation

**Fig. 14:**
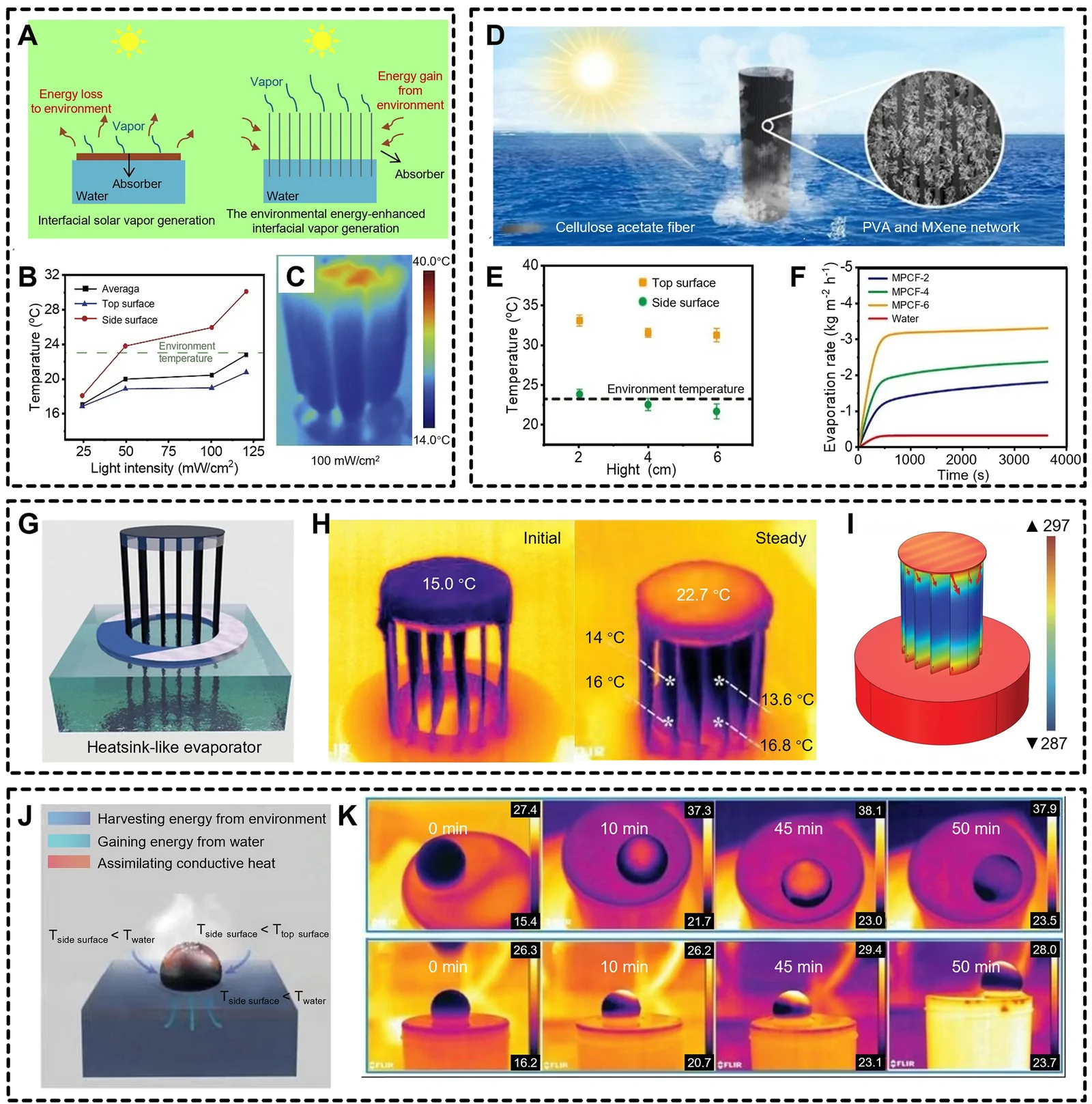
**A** Comparison between SDIE and environmental energy-enhanced SDIE. **B** Surface temperature variations on the top and lateral faces of the environmental energy-enhanced SDIE. **C** IR imaging results of the environmental energy-enhanced SDIE under 100 mW cm.^−2^ illumination [3]. **D** Schematic illustration of MXene/PVA modified the cigarette filter (MPCF) SDIE. **E** Measured surface temperatures at the top and sidewalls of MPCFs with differing exposed heights under standard 1 sun. **F** Comparison of water evaporation rates of pure water and various MPCF configurations (2, 4, and 6) under 1sun exposure [123]. **G** Scheme of the heatsink-like (HSE). **H** Infrared images of the 6-fin HSE captured at the initial stage and thermal steady state following 30 min of 1-sun illumination. **I** Modeled temperature distribution and heat flow trajectories on the evaporator during photothermal evaporation [41]. **J** Scheme illustrating the energy-coupling mechanisms operative in the dual-zone photothermal sphere during solar-driven evaporation. **K** Time-lapsed IR imaging and side view of the photothermal sphere under standard solar illumination [124]

SDIE operates through three primary energy transfer mechanisms: solar energy absorption, steam generation, and thermal exchange. Traditional strategies aimed at enhancing the evaporation efficiency have primarily focused on maximizing solar absorption while mitigating heat loss to the environment. However, these approaches typically elevate the absorber temperature above ambient levels, leading to inevitable thermal losses to the surroundings (Fig. 14A). Under these conditions, only a fraction of incident solar energy is converted into internal steam energy, with the remainder lost as thermal dissipation, thereby reducing the solar-to-steam energy conversion efficiency to less than 100%. By contrast, if the absorber temperature can be maintained below ambient conditions, the system may draw additional energy from the surrounding environment, which enables to substantially increase the evaporation rate, potentially surpassing the theoretical limit.

For example, Li et al. [3] utilized cotton cores as one-dimensional water supply pathways to minimize heat conduction losses to the underlying bulk water. A hydrophilic cellulose layer with hierarchical, interconnected pores facilitated continuous vapor release and air infiltration. Vapor generation induces heat absorption from the cotton core, cooling the sidewall of evaporator below ambient temperature (Fig. 14B, C). In this state, the evaporator sidewall absorbs environmental heat through convection and radiation. Consequently, the evaporation rate of the cotton core evaporation array under one sun (1.62 kg m^−2^ h^−1^) exceeded the theoretical evaporation maximum (1.47 kg m^−2^ h^−1^) for 100% photothermal conversion efficiency. Similarly, Li et al. [123] fabricated an environmentally enhanced SDIE by processing cellulose acetate filters through PVA solution immersion, lyophilization, and MXene deposition (Fig. 14D). As the height of the evaporator side walls increases, the temperature of the side walls gradually decreases (Fig. 14E), which enhanced environmental energy harvesting, thereby boosting evaporation rates (Fig. 14F). Wu et al. [41] further advanced this concept by designing a heatsink-inspired evaporator (HSE) with radial fin arrays (Fig. 14G). The solar evaporation surface temperature fell below ambient levels in evaporators featuring 5–7 heatsink fins (Fig. 14H, I), effectively eliminating radiative, convective, and conductive losses. This “cold evaporation” mechanism achieved a record rate of 4.32 kg m⁻^2^ h⁻^1^ under 1 sun. Despite these breakthroughs, salt accumulation on sub-ambient evaporation surfaces remains a critical bottleneck for sustained operation. Addressing this, Wu et al. [124] developed a spherical evaporator (Fig. 14J) featuring a photothermal core that establishes a localized cold evaporation zone (Fig. 14K), coupled with self-rotation driven by asymmetric mass distribution. This dual-function design enabled continuous salt rejection via dynamic re-dissolution, maintaining a high evaporation rate of 2.06 kg m⁻^2^ h⁻^1^ over 8 h in 20 wt% NaCl solution. A comprehensive summary and comparative analysis of the reported works have been meticulously conducted (as shown in Table 1).

**Table 1:** The lignocellulosic biomass materials, functions, construction strategies, light absorption, surface operating temperature, and evaporation rates of lignocellulosic biomass-based SDIEs

Lignocellulosic biomass	Function	Construction strategy	Light absorption	Surface operating temperature (°C)	Evap. rate (kg m^−2^ h^−1^)	Refs
Wood + lignin	Substrate + photothermal	Coating + delignification	> 95% [300–2500 nm]	38.2	2.89	[34]
Wood	Substrate	Delignification	> 97.9% [300–2500 nm]	43	2.82	[79]
Lignin	Photothermal	Carbonization	≈90% [200–2500 nm]	60.4	1.539	[80]
Wood	Substrate	–	≈91.5% [200–2500 nm]	38	1.2	[81]
Wood	Photothermal + substrate	Pressure-assisted carbonization	≈97% [200–2500 nm]	32	6.4	[87]
Wood	Photothermal + Substrate	Pressure-assisted carbonization	–	44.7	1.35	[88]
Wood	Photothermal + substrate	Scanning flame treatment	–	33	1.3	[35]
Wood	Photothermal + substrate	Laser engraving	≈95% [200–2500 nm]	49.4	1.72	[89]
Wood	Substrate	Delignification	≈90% [200–2500 nm]	37.8	1.94	[93]
Cellulose + lignin	Substrate	Blending	> 90% [200–2500 nm]	28.7	1.6	[94]
Cellulose	Substrate	Blending	≈96.8% [300–2500 nm]	43.1	3.02	[36]
Wood	Substrate	Delignification	> 95% [300–2500 nm]	42.1	2.08	[9]
Wood	Substrate	Delignification	–	43.8	2.04	[37]
Lignin	Photothermal	Carbonization	≈95.5% [200–2500 nm]	44	2.351	[98]
Wood	Substrate	Delignification	> 96.85% [250–2500 nm]	72.6 [dry]	1.927	[102]
Wood	Substrate	Remove lignin and hemicellulose	> 95% [250–2500 nm]	44.4	1.394	[103]
Wood	Substrate	Delignification	≈99% [250–2500 nm]	30.6	–	[1]
Wood + lignin	Substrate + photothermal	Delignification	–	31	1.18	[104]
Cellulose	Substrate	Directional freezing	≈97.3% [300–2500 nm]	39.3	3.2	[113]
Cellulose	Substrate	Directional freezing	≈95.8% [200–2500 nm]	51.2	2.287	[45]
Cellulose	Substrate	Directional freezing	≈94% [200–2500 nm]	57	2.034	[114]
Lignin	Tunes water state	Blending	≈95% [300–2500 nm]	47.1	2.09	[77]
Cellulose	Substrate	3D Print	≈97% [300–2500 nm]	41.7	1.33	[115]
Cellulose	Substrate	3D Print	> 97% [250–2500 nm]	48.7 [3 sun]	0.97	[44]
Cellulose + lignin	Substrate	Directional freezing	≈91% [250–2500 nm]	–	1.29	[38]
Cellulose	Substrate	Freeze-drying	≈95% [250–2500 nm]	38	1.82	[7]
Cellulose	Substrate	Blending	≈93.7% [200–2500 nm]	31	1.15	[6]
Wood	Substrate	–	–	75.7 [3.5 sun]	1.38	[120]
Wood	Substrate	Gelation	≈90.81% [300–2500 nm]	35.1	1.92	[121]
Cellulose	Substrate	Freeze-drying	≈93.72% [200–2500 nm]	35	1.9	[39]
Cellulose	Substrate	–	≈96% [200–1100 nm]	26.6	1.62	[3]
Cellulose	Substrate	Blending	≈95% [250–2250 nm]	31.2	3.38	[123]
Cellulose	Substrate	Freeze-drying	> 95% [250–800 nm]	22.7	4.1	[41]
Cellulose	Substrate	Blending	> 95% [250–800 nm]	50 [dry]	2.6	[124]

### Light Absorption

**Fig. 15:**
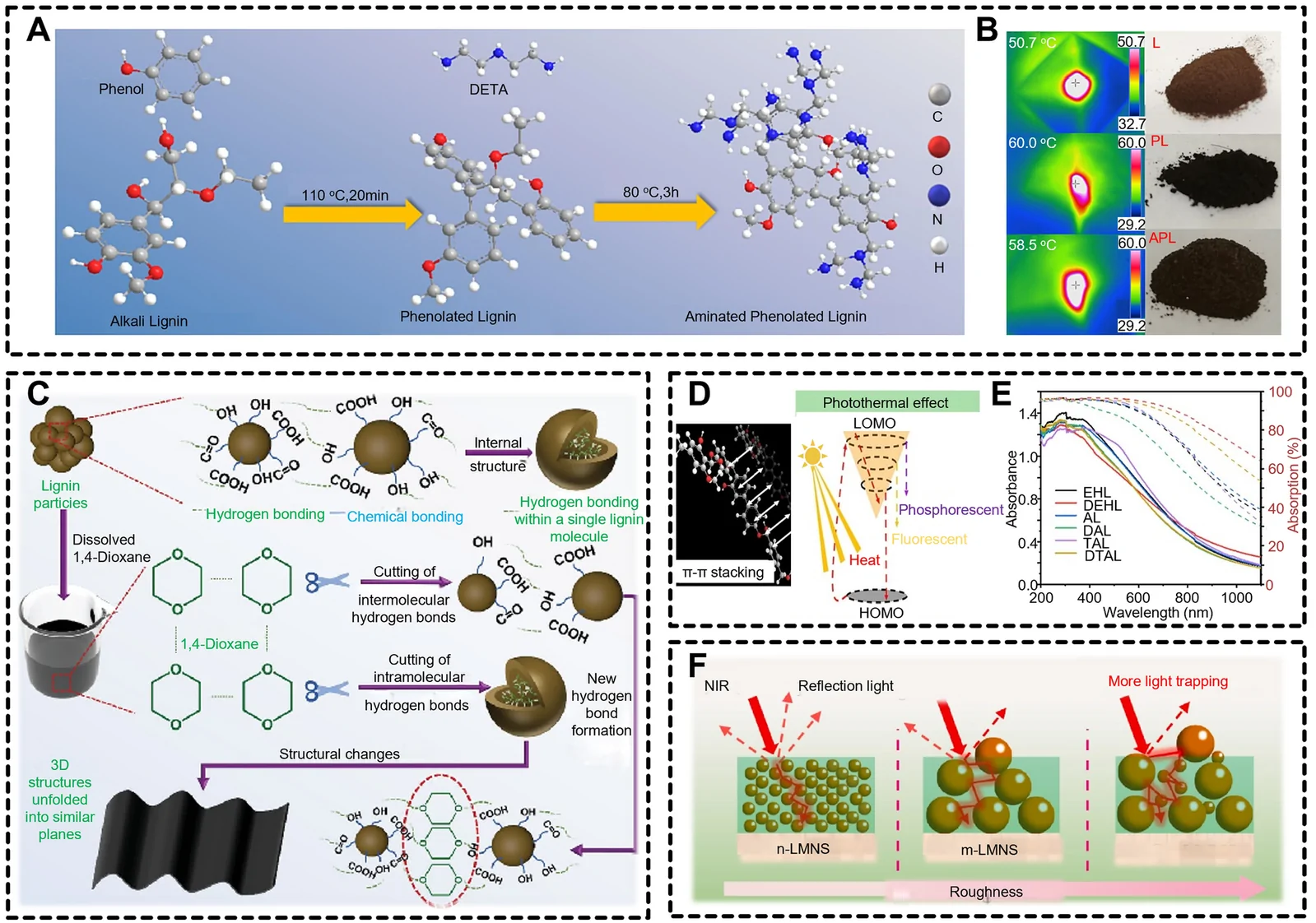
**A** Illustrative schematic of the modification process of lignin. **B** Infrared thermal images of alkali lignin (L), phenolated lignin (PL), and aminated phenolated lignin (APL) under 1-sun irradiation [125]. **C** Schematic representation of the structural changes and associated changes in conjugated interactions of lignin particles before and after dissolution [12]. **D** The enhanced photothermal effect of lignin. **E** Ultraviolet spectra (solid lines) and corresponding light absorption profiles (dashed lines) of different lignin samples [40]. **F** Representation of the light trapping efficiency and reflective responses in coatings with varying surface textures, composed of lignin nanospheres (n–LMNSs), microspheres (m–LMNSs), and dual-sized micro-nanospheres (m@n–LMNSs) [126]

The UV absorption of lignin capacity derives from its abundant UV absorbing chromophore groups, including conjugated phenols, ketones, quinone structures, and intramolecular hydrogen bonds [127]. Therefore, lignin mainly absorbs ultraviolet light. Researchers confirm that lignin aggregation induces the formation of aromatic stacking and *π*–*π* conjugated systems [128]. These results establish the photothermal conversion capability of lignin [75], supporting its potential for solar energy applications. This chapter summarizes the methods—increasing *π*–*π* conjugation, increasing *π*–*π* stacking, and physical processing—for improving the light absorption capacity of lignin. The photothermal conversion efficiency of lignin can be significantly enhanced by increasing *π*-orbital conjugation or hyperconjugation. This modification reduces the electronic bandgap, promoting more efficient light absorption, particularly in the infrared region. Nonradiative decay minimizes energy losses via fluorescence or phosphorescence and maximizes heat generation. Chemical functionalization approaches, such as phenolation and acetylation, offer a robust route to enhance *π*–*π* conjugation within lignin. Yue et al. [125] enhanced the photothermal conversion efficiency of lignin by grafting additional benzene rings through an acid-catalyzed condensation reaction with phenol at 110 °C (Fig. 15A). Under acidic conditions, protonation-induced carbocation formation on the lignin side chain facilitated C–C bond formation with phenol, leading to the development of stable polycyclic *π*-conjugated systems [129]. This modification disrupted weak chemical bonds within lignin, such as ether linkages, generating new active sites and further enhancing phenolization [130]. PL and APL attain surface temperatures of approximately 60 °C, significantly higher than that of unmodified lignin, indicating enhanced photothermal conversion efficiency through modification (Fig. 15B). Lei et al. [131] demonstrate that acetyl functionalization of lignin induces the formation of robust electron donor–acceptor (D-A) conjugated systems, reducing the bandgap and enhancing light absorption. Under 808-nm laser irradiation (0.51 W cm⁻^2^), the acetylated lignin exhibits exceptional photothermal performance, reaching a maximum surface temperature of ≈175 °C with a photothermal conversion efficiency of 73.2%. A comparable donor–acceptor architecture can be referenced in studies pertaining to melanin [132].

Carbonization and solvent dissolution are two effective strategies for modulating *π*–*π* conjugation in materials such as lignin. Lin et al. [10] subjected lignin to carbonization to obtain lignin-derived carbon. The presence of the pore-forming agent (KOH) facilitates the formation of numerous pores induced by erosion. Its porous structure and high surface area prompt 98% full-spectrum solar absorption [133]. Another innovative strategy to promote *π*–*π* conjugation and enhance photothermal conversion involves dissolving lignin in specific solvents, such as 1,4-dioxane. Gu et al. [12] proposed a mechanism explaining the changes in conjugate intensity before and after lignin dissolution (Fig. 15C). The dissolution process in 1,4-dioxane significantly enhanced the light absorption of lignin by disrupting hydrogen bonds, leading to a more ordered structure and increased exposure of conjugated structures. This reduced structural obstructions and allowed more photons to be absorbed, improving energy capture. As a result, the dissolved lignin exhibited significantly higher absorption in the 500–2000 nm range compared to solid lignin powder.

Optimizing *π*–*π* stacking interactions in lignin architectures significantly enhances photothermal conversion ability. This approach leverages the inherent aromaticity of lignin, quenching aggregation-induced luminescence and promoting nonradiative relaxation, which leads to more efficient heat generation [131, 134]. Given the substantial methoxy group content in lignin, particularly in hardwoods, demethylation has emerged as an effective strategy to enhance the phenolic hydroxyl content. This modification reduces steric hindrance, thereby promoting intermolecular interactions. In addition to facilitating *π*–*π* stacking, demethylation strengthens hydrogen bonding and van der Waals forces, leading to significant improvements in both photothermal conversion efficiency and thermal response. For example, Shao et al. [40] demonstrated that the activation of lignin using iodocyclohexane (ICH) disrupts its molecular structure and reduces the methoxy content, thereby enhancing intermolecular bonding. Phenolic hydroxyl hydrogen bonding enhances *π*–*π* stacking interactions among benzene rings (Fig. 15D). UV–Vis–NIR spectroscopy reveals enhanced light absorption across the spectrum for modified versus unmodified lignin samples (dashed line) (Fig. 15E). And, Zhao et al. [135] removed the methyl groups from lignin (D-Lig) and subsequently coordinated the resulting D-Lig with Fe^3^⁺ to generate D-Lig-Fe, which exhibited further enhanced photothermal conversion. Liu et al. [136] also demonstrated that copper-ion coordination with alkali lignin induces high broadband absorption across the solar spectrum.This approach aligns with the established utility of metal-catechol coordination for crafting functional materials, a strategy gaining increasing traction [137].

Beyond increasing *π*–*π* conjugation and increasing *π*–*π* stacking, nanoscale processing and preparation of composite materials serve as critical physical strategies to enhance lignin photothermal performance. The conversion of lignin into nanoparticles through nanotechnology significantly enhances its specific surface area, thereby improving photothermal conversion efficiency. Ma et al. [126] fabricated a photothermal superhydrophobic coating using dual-scale lignin micro-nanospheres (micro-LMNSs and nano-LMNSs) (Fig. 15F). Upon laser irradiation, the photothermal effect led to a rapid surface temperature increase from approximately 13 to 112 °C within 60 s, indicating excellent photothermal responsiveness. Furthermore, combining lignin with materials such as graphene, porous carbon, and metals facilitates the creation of composite photothermal materials. Shao et al. [138] prepared a lignin-guided solution containing copper sulfide (CuS) nanoparticles, and the polyvinyl alcohol (PVA) photothermal film fabricated from this lignin-stabilized CuS solution exhibited remarkable solar absorption (≈ 95%) and uniform dispersion of CuS nanoparticles. This film demonstrated a photothermal conversion efficiency of approximately 49.43%, presenting a novel strategy for the synthesis of metal nanoparticles stabilized by lignin.

While substantial progress has been made in improving the photothermal conversion efficiency of lignin, the influence of its structural and compositional characteristics—such as the nature and concentration of functional groups, lignin subclass, aromaticity index, and molecular weight—on conjugation effects, *π*–*π* stacking, and overall photothermal properties remains inadequately understood. Therefore, further detailed investigations are required to fully elucidate the relationship between these structural factors and the photothermal behavior of lignin. Moreover, the use of lignin as a photothermal material in solar-driven interface evaporators remains underexplored.

### Water Management

#### Water Transport

**Fig. 16:**
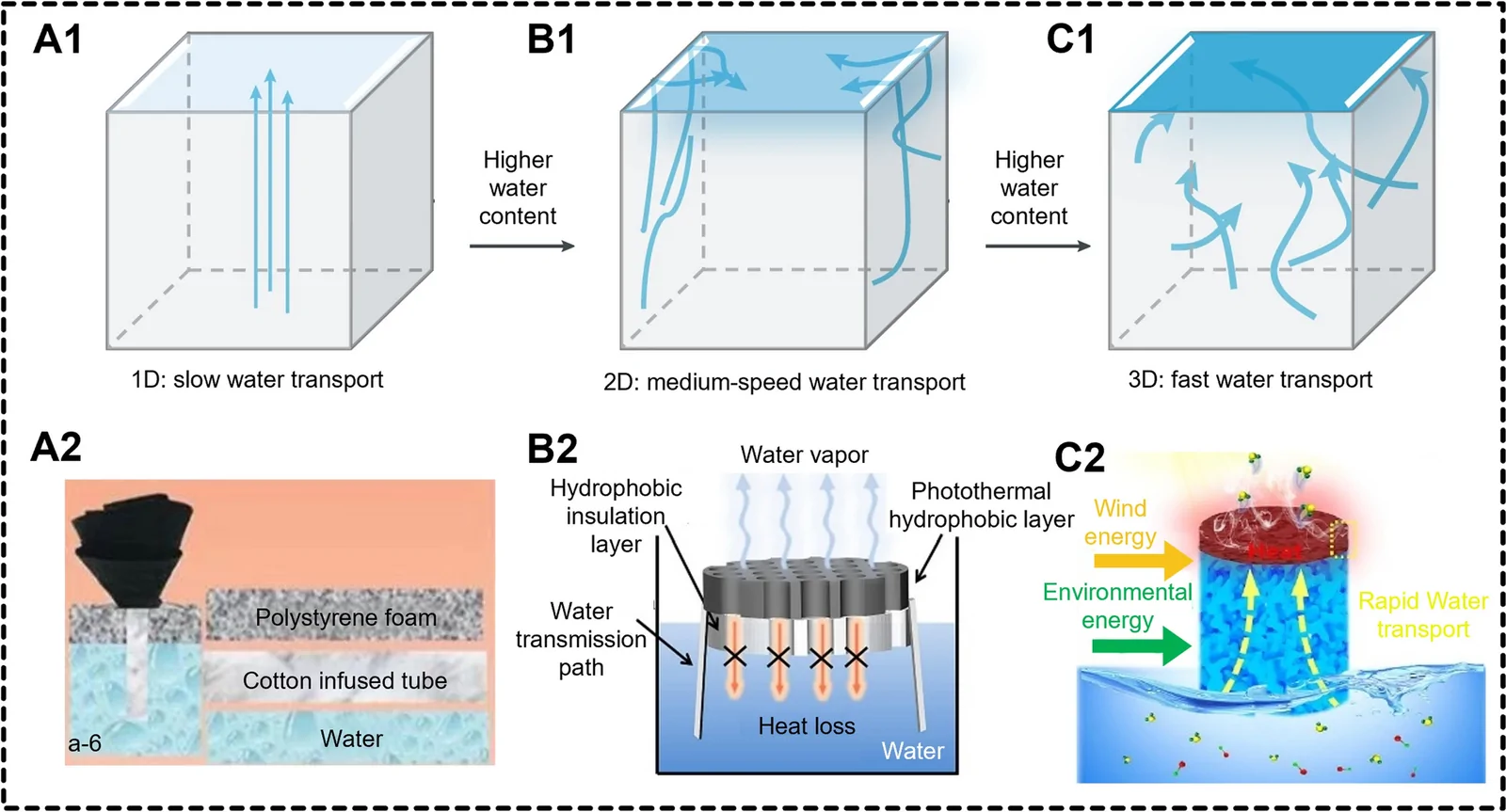
**A1**–**C1** 1D, 2D and 3D waterway design [42], and corresponding examples **A2**) [139] **B2**) [140] **C2**) [141]

Efficient water transport pathways are essential for ensuring the continuity of the photothermal evaporation process in SDIEs. According to existing studies, transport channels in these systems are classified by their dimensionality, including 1D, 2D, or 3D (Fig. 16A–C), and different dimensional structures are used to control water transport speed and tune water content. 1D water pathways are primarily designed to minimize heat loss by directing water through a narrow, singular channel, thereby enhancing the efficient utilization of thermal energy (Fig. 16A). Li et al. [139] employed a cotton tube as a 1D water path, effectively reducing heat losses through convection, conduction, and radiation. However, the low rate of water transport may not be sufficient to match the evaporation rate in cases where the evaporation area is large, potentially constraining purified water yield per unit of time. In contrast, 2D water paths adopt physical isolation between thermal insulation and hydraulic conduits to mitigate conductive heat loss (Fig. 16B). Typically, water is restricted to the sides of the insulation layer. For instance, Hu et al. [140] utilized a hydrophobic cellulose/TiN aerogel photothermal layer paired with a dual-channel air-laid paper for hydraulic mediation. The water transport rate is enhanced with an increase in the capillary strength of the side-wrapping material.

To further enhance evaporation performance, 3D SDIEs with interconnected porous structures have been developed (Fig. 16C). However, the extensive heat exchange between water and air at high porosities can lead to significant heat loss. Therefore, it is essential to optimize porosity and channel configuration of water transport materials when designing SDIEs to ensure an efficient balance between evaporation energy and water flux. To address this challenge, Li et al. [141] developed a 3D bilayer evaporator by incorporating a superabsorbent polymer, polyvinyl alcohol phosphate ester (PVAP), into a CNF aerogel matrix, with CNTs serving as the light-absorbing layer. This design not only facilitates efficient solar absorption but also harnesses environmental energy through convective and radiative heat transfer, enhancing the overall evaporation rate. Moreover, the nanoscale dimensions and polar surface groups of CNFs confer high hydrophilicity and tailored porous architecture, facilitating efficient water transport. The interplay between polymer crosslinking density, pore size, and water transport remains contentious. While crosslinking typically governs mechanical robustness, its role in hydraulic dynamics is less clear [142]. For instance, Li et al. [143] observed that narrower channels, compared to larger pores, exhibit stronger capillary action. In contrast, Mao et al. [144] observed accelerated transport in macroporous frozen gels relative to dense hydrogels. This inconsistency highlights a knowledge gap regarding the potential effects of crosslinking density and pore size on water transport kinetics in SDIEs.

#### Regulation of Water Sate

**Fig. 17:**
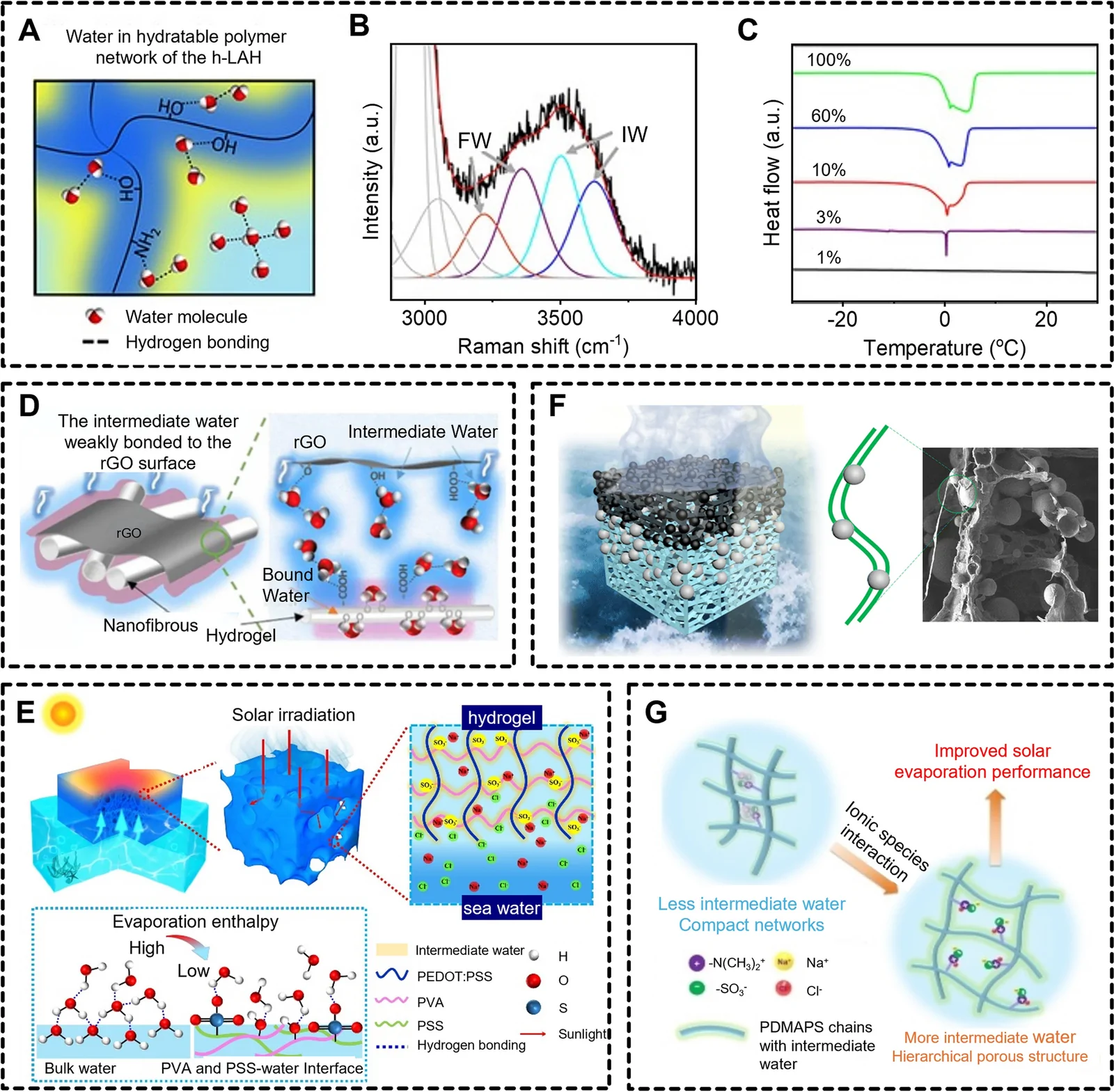
**A** Schematic illustration of water distribution within the hydratable polymer network, showing water/polymer bonding, weakened water/water bonding, and normal water/water bonding. **B** Raman spectra illustrating the fitting peaks representing intermediate water (IW) and free water (FW). **C** Differential scanning calorimetry (DSC) curves of the hydratable light-absorbing hydrogel (h-LAH) with varying hydration levels [43]. **D** Schematic illustration of the interfacial evaporation of water from the hybrid nanofibrous hydrogel-reduced graphene oxide (NHrG) membrane [145]. **E** Schematics of mechanisms about enhanced SDIE performance [146]. **F** Graphical and SEM representations of a cellulose evaporator with a gradient in wettability properties [147]. **G** Schematic representation of solar steam generation based on a hydrogel composed of salt-tolerant anion polyelectrolytes [148]. (Color figure online)

The hydration state of water in SDIE systems is governed by distinct hydrogen-bonding regimes: bound water (BW), intermediate water (IW), and free water (FW), categorized by their molecular interactions (Fig. 17A). FW (light blue), located distal to hydrophilic matrices, exhibits bulk-like behavior with four hydrogen bonds per molecule, necessitating significant energy for evaporation. BW (dark blue), tightly bound to polar polymer chains via strong hydrogen bonds, demands the highest evaporation enthalpy. IW (yellow), situated between BW and FW, forms weaker hydrogen bonds with fewer than four neighbors, enabling lower-energy evaporation. Modulating these hydration states, particularly enhancing IW, can reduce the overall enthalpy of evaporation, a critical lever for optimizing SDIE efficiency. Experimental validation of IW relies on differential scanning calorimetry (DSC) and Raman spectroscopy. Raman spectra of hydrated systems reveal hydrogen-bonding states: Peaks at 3233 and 3401 cm⁻^1^ correspond to tetrahedrally coordinated FW, while 3514 and 3630 cm⁻^1^ reflect weakly bonded IW (Fig. 17B). DSC thermograms further distinguish nonfreezable BW (no phase-change signal) from freezable IW and FW. Fully hydrated systems exhibit dual endothermic peaks at 0 °C (IW melting) and ~ 5 °C (FW melting), whereas dried samples lack these signals (Fig. 17C). Critically, IW generation depends on hydratable polymer networks, independent of total water content (purple curve), highlighting the role of matrix chemistry in hydration dynamics.

Strategies to amplify IW focus on tuning polymer–water interactions. For example, Zang et al. [145] reported that graphene oxide (GO)-enhanced hydrogels introduce oxygenated functional groups, forming weak hydrogen bonds that preferentially stabilize IW (Fig. 17D). Similarly, Li et al. [146] demonstrated that increasing the hydrophilic group density results in a considerable rise in the IW content (Fig. 17E). However, excessive hydrophilicity risks BW accumulation due to strong polar interactions, highlighting a trade-off between IW promotion and energy-intensive BW formation. To mitigate this, recent work explores inhomogeneous wettability—heterogeneous surface chemistry engineered via doping or polymerization. Sun et al. [147] embedded hydrophobic SiO_2_ spheres into cellulose aerogels, creating localized hydrophobic domains that weaken cellulose–water interactions while retaining IW-rich regions (Fig. 17F). The aerogel design achieved a thermal conductivity of 0.1008 W m^−1^ K^−1^ in the wet state while maintaining heterogeneous wettability, which reduced cellulose–water interactions and increased the depinning force at the evaporative contact line. A persistent challenge remains salt deposition during high-rate evaporation. As shown in Fig. 17G, Lei et al. [148] harnessed the anti-polyelectrolyte effect to engineer a class of polyzwitterionic hydrogels (PZHs) with enhanced SDIEs performance in high-salinity brines. PZHs contain oppositely charged cationic and anionic groups distributed along their polymer backbones [149]. Brine ions attenuate interchain electrostatic attraction, inducing polymer chain expansion and increased water absorption—the anti-polyelectrolyte effect [150]. The resulting hydrated polymer network exhibits increased IW content, enhancing evaporative performance.

## Multifunctional Integrated Applications of SDIEs

### Desalination

Salt crystallization at the evaporation interface poses a critical challenge for solar-driven desalination systems, where rapid water vaporization concentrates solutes, leading to surface salt deposition that obstructs light absorption, blocks water channels, and degrades long-term performance [151, 152]. To address this, recent innovations focus on structural and material engineering to redirect or dissolve salts, including asymmetric wettability engineering [11], dynamic crystallization control [153], and pore architecture optimization [154].

**Fig. 18:**
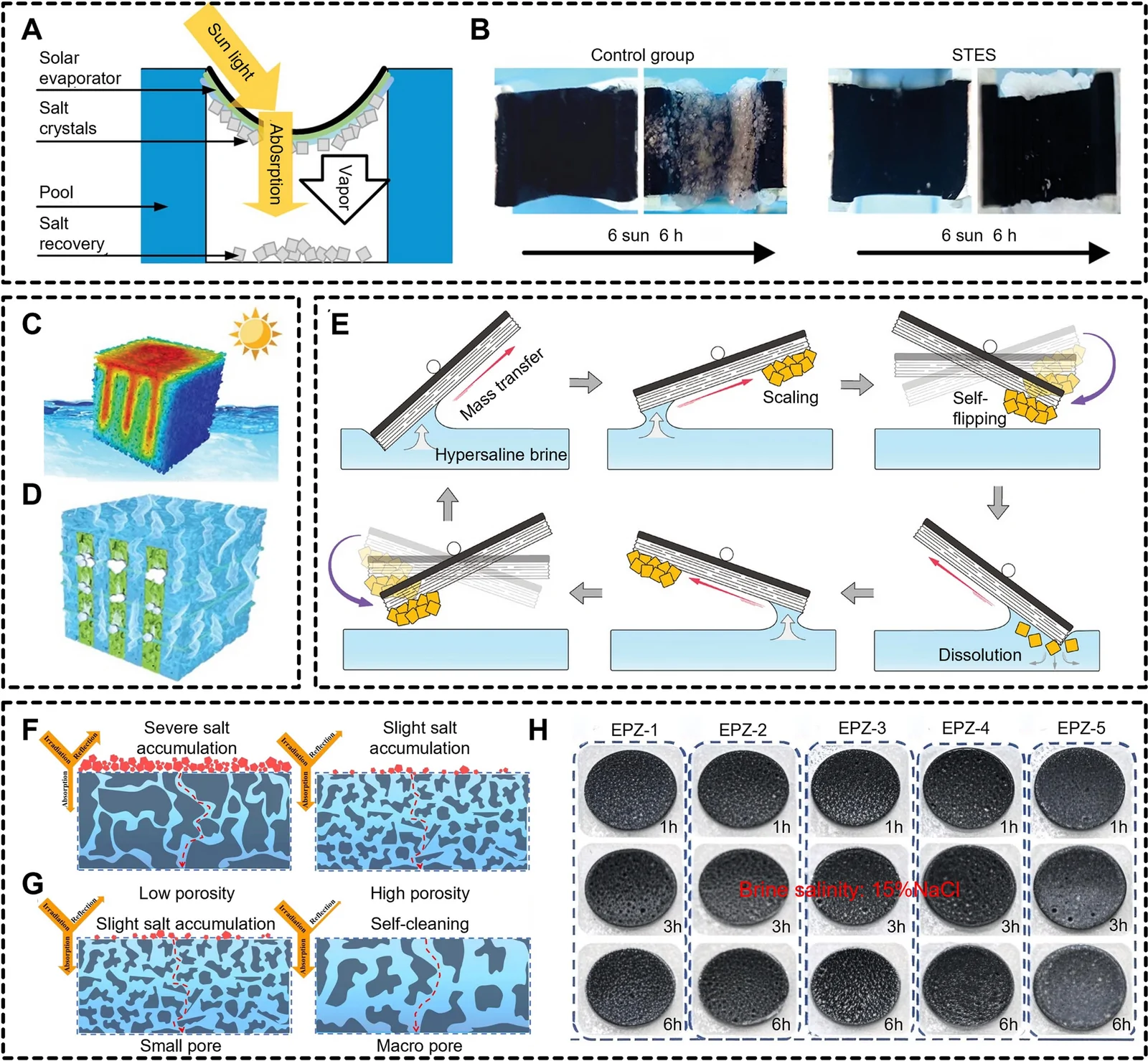
**A** Illustrative diagram depicting the mechanisms of suspended-type evaporators (STEs).** B** Salt crystallization phenomena observed on the photothermal layer of the control group and STEs [11]. **C** Schematic of the 3D evaporators for solar desalination. **D** Schematic of the directional salt crystallization [153]. **E** Cyclical self-flipping processes [155]. **F** Diagram illustrating the role of porosity in modulating salt accumulation. **G** Conceptual illustration showing the relationship between pore size and salt accumulation. **H** Time-dependent changes in the crystallization of salt on evaporators with distinct pore sizes [154]

A prominent approach involves designing evaporators with asymmetric wettability to spatially separate salt crystallization zones from active evaporation interfaces. For instance, Dong et al. [11] introduced a suspended-type evaporator (STE) constructed from Janus fibrous mats (Fig. 18A). The fibrous structure efficiently wicks brine into the evaporation layer, where salt is retained until crystallization. The suspended design ensures zero liquid discharge. This design confines salt crystallization to the hydrophilic underside while maintaining a hydrophobic photothermal interface, achieving a sustained evaporation rate of 1.94 kg m⁻^2^ h⁻^1^ and enabling full mineral recovery (Fig. 18B). Similarly, Wang et al. [153] reported a metal–phenolic network (MPN)-engineered 3D evaporator with alternating superhydrophilic/superhydrophobic sponges coated in metal–phenolic networks (MPNs) and side-twining hydrophilic threads (Fig. 18C). The threads directed salt ions toward designated crystallization sites, preventing surface fouling while maintaining a high desalination rate of ≈ 2.3 kg m⁻^2^ h⁻^1^ in 20 wt% brine under 1-sun irradiation (Fig. 18D).

In addition to promoting the directional crystallization of salts, the structural design of the evaporator enables the self-cleaning of salt deposits through the crystallization process. This approach not only enhances the operational efficiency of the system but also reduces the need for manual intervention, thereby improving the long-term sustainability of the evaporator. Chen et al. [155] reported a Janus-structured seesaw evaporator that utilizes scaling for autonomous descaling. The evaporators are fabricated through a two-step process: delignification of balsa wood followed by single-sided application of soot and PDMS coatings. The unique Janus architecture ensures continuous solution supply during evaporation while maintaining buoyancy on saline water. Evaporation induces directional salt ion transport to the elevated evaporator terminus, enabling localized scaling. Once the accumulated salt reaches a critical mass, the seesaw mechanism triggers a flip, causing the salt to dissolve back into the solution (Fig. 18E). Under 8 wt% saline conditions, the system demonstrates a water evaporation rate of 2.65 kg m^−2^ h^−1^.

Concurrently, internal pore structure optimization offers a complementary strategy to mitigate salt accumulation. Huang et al. [154] fabricated a porous evaporator with bimodal pore sizes (150–300 μm macropores and < 10 μm micropores) and 70.4% porosity. The macroporous network reduced hydraulic tortuosity by 62%, shortening salt ion back-diffusion paths, while micropores enhanced capillary pumping (Fig. 18F, G). This architecture enabled continuous salt rejection in 15% NaCl brine, maintaining a stable rate of 1.90 kg m⁻^2^ h⁻^1^ under 1.5-sun irradiation (Fig. 18H). Quantitative analysis revealed a 78% reduction in surface salt coverage compared to low-porosity (45%) counterparts, directly linking pore geometry to anti-fouling performance.

### Energy Generation

The integration of multifunctional components into SDIE systems has expanded their applications, particularly in concurrent power generation, a synergy aligning with the core principles of energy conservation and environmental sustainability. These hybrid systems leverage environmental energy gradients to enhance overall efficiency while addressing broader decarbonization goals. Current power generation strategies in SDIEs primarily exploit two energy sources: (1) process-inherent energy (thermal or salinity gradients generated during evaporation) and (2) ambient environmental energy (e.g., wave, wind) [157, 158].

**Fig. 19:**
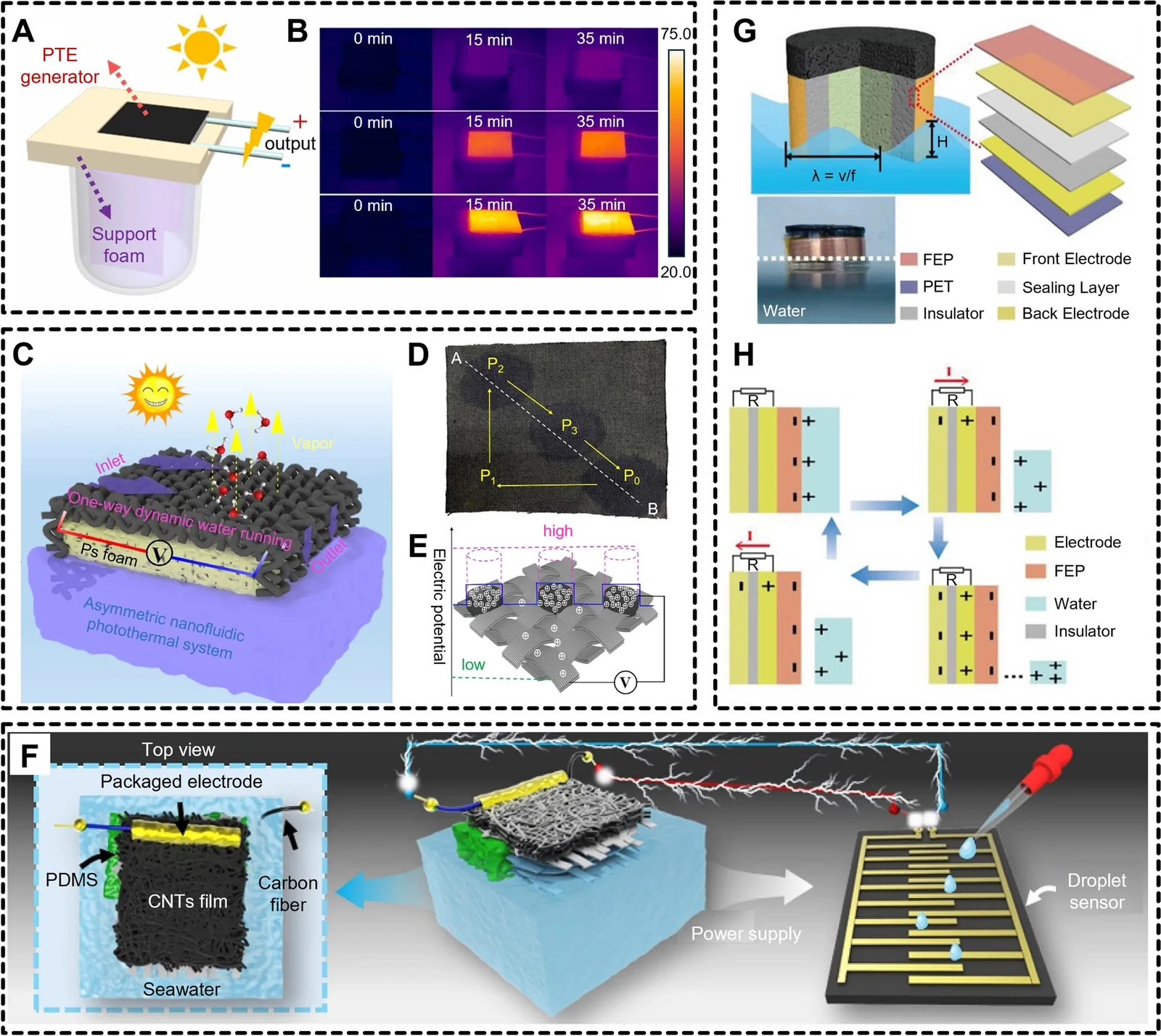
**A** Diagram of photo-thermal-electro (PTE) generator. **B** Infrared images illustrating the surface temperature of the bare generator, PTE generator under 1 sun, and the bare generator under 2 sun at specific time points (0, 15, and 35 min) [38]. **C** Schematic depiction of a plant-inspired asymmetric nanofluidic photothermal system for the dual purposes of solar desalination and electrokinetic power generation. **D** Photograph of the MXene/cotton textile. **E** Schematic illustration of the potential difference observed between the dense core and regions with low MXene concentration under the drenching state [156]. **F** A schematic demo of self-powering sensor [6]. **G** Illustrative diagram and a digital photograph of integrated SDIE and triboelectric nanogenerator (TENG). **H** Operational principle of TENG [7]

Thermoelectric conversion capitalizes on temperature differentials between evaporative interfaces and bulk water. For example, the STA-EGaIn cellulose aerogel was developed by Wei and colleagues [38] that attained a surface temperature of 56.2 °C under 1-sun illumination, demonstrating efficient photothermal conversion. This significant temperature differential between the high-temperature evaporation surface and the low-temperature bulk water enabled the generation of electricity via the Seebeck effect (Fig. 19A, B). Coupled with p-type thermoelectric (PTE) modules, this configuration generated 74.43 mV open-circuit voltage and 5.77 mA short-circuit current. However, thermoelectric power generation necessitates maintaining the evaporator surface at an elevated temperature, which presents notable challenges. High surface temperatures can result in increased thermal radiation and convection, leading to inefficiencies through unnecessary heat loss from the evaporation system. Furthermore, under realistic conditions, few photothermal materials are capable of sustaining long-term thermal stability that is a critical bottleneck for scalability.

The salinity gradient between seawater and freshwater holds a significant energy potential [159]. This blue energy (renewable energy) potential exists within all evaporative systems. To maximize charge separation from salinity gradients and optimize blue energy harvesting, researchers have developed specialized structures including directional water transport systems [160] and unilateral salinity accumulation Nafion membrane structure [161]. Peng and co-workers [156] developed an MXene-cotton evaporator with diagonal nanosheet deposition (Fig. 19C), creating spatially segregated cation (H_3_O^+^/Na^+^) and anion (OH^−^/Cl^−^) zones (Fig. 19D). This asymmetry established electric double layers (EDLs) at wetting interfaces, yielding 363 mV voltage output from 3.5 wt% brine under 1 sun (Fig. 19E). Similarly, Xiao et al. [6] achieved unidirectional proton transport via plasma-treated CNT membranes on asymmetric cellulose paper (Fig. 19F), attaining 2.1 μW power output through evaporation-enhanced water potential gradients. While effective, such designs introduce mechanical fragility, the added functional layers reduce evaporator tensile strength by 40–60% compared to monolithic structures, posing durability trade-offs. Environmental energy harvesting further diversifies functionality. Li et al. [7] integrated triboelectric nanogenerators (TENGs) onto cellulose aerogel sidewalls (Fig. 19G), converting omnipresent wave energy into electrical signals for real-time water quality monitoring (Fig. 19H). This dual-function system maintained 4.32 kg m⁻^2^ h⁻^1^ evaporation rates while generating actionable environmental data, a paradigm for smart water management.

Despite progress, critical challenges persist: 1) Energy-form competition: Maximizing one energy output (e.g., electricity) often compromises another (e.g., evaporation rate); 2) Material compatibility: Heterogeneous component integration risks interfacial delamination under cyclic thermal/hydraulic stresses; 3) Scalability: Laboratory-scale ion gradient amplification (e.g., unilateral Nafion membranes) struggles to translate to meter-sized systems. Future designs must adopt holistic optimization frameworks, balancing multi-energy output ratios with structural robustness across varying operational scales.

### Wastewater Treatment and Anti-microbial

**Fig. 20:**
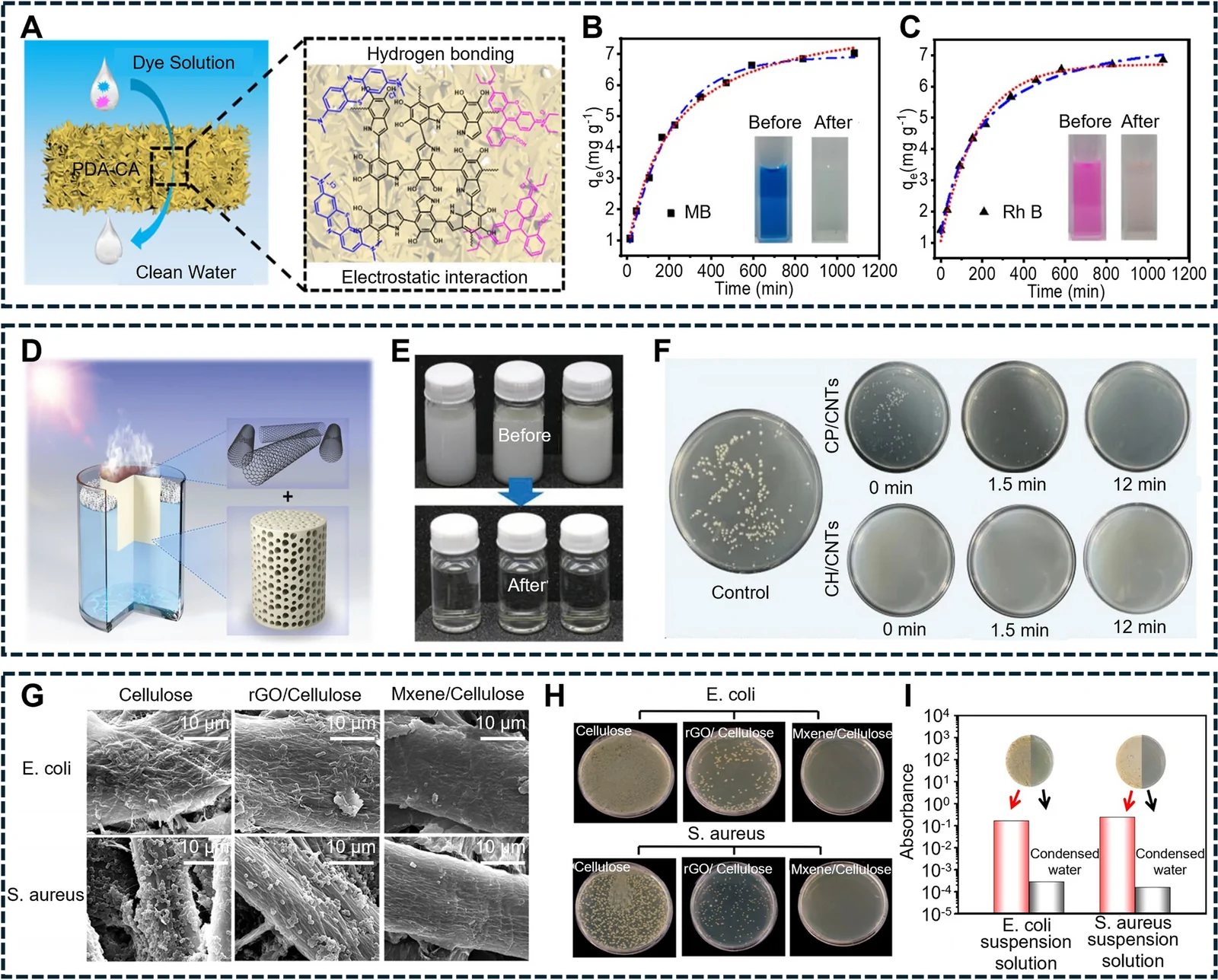
**A** Graphical representation of dye removal via the polydopamine-enriched cellulose aerogel (PDA-CA) through physical adsorption. **B**, **C** Kinetic adsorption characteristics of PDA-CA were analyzed using pseudo-first-order (red dotted line) and pseudo-second-order (blue dotted line) models for curve fitting. The inset demonstrates the dye removal capacity of PDA-CA [162]. **D** Schematic diagram of the corncob pith/carbon nanotubes (CP/CNTs) evaporator. **E** Microemulsion oily water before and after purification. **F** Photographs of bacterial colonies, cultured alone and co-cultured with CP/CNTs or cellulose hydrogel/carbon nanotubes (CH/CNTs), under a single exposure to sunlight for 0, 1.5, and 12 min [163]. **G** SEM images of the different membrane surfaces with adhered bacteria. **H** Digital images of E. coli and S. aureus in contact with cellulose, rGO/cellulose, and MXene/cellulose membranes for a 24-h period, respectively. **I** UV–Vis absorbance measurements of E. coli and S. aureus suspensions, taken before and after purification. The corresponding digital images show the surfaces of solid nutrient agar plates, with E. coli suspension on the left and S. aureus suspension on the right [5]. (Color figure online)

SDIEs also exhibit significant application potential in the field of wastewater treatment. Dye adsorption has been engineered through molecular interaction tailoring. Zou et al. [162] designed polydopamine-filled cellulose aerogel (PDACA) featuring synergistic binding sites (Fig. 20A). The catechol/quinone groups in polydopamine (PDA) form hydrogen bonds with amine/sulfonic moieties in dyes, while *π*–*π* stacking between aromatic systems and electrostatic attraction further enhances capture [164, 165]. This multi-modal adsorption achieved excellent removal efficiency for methylene blue (11.5 mg g^−1^) within 60 min (Fig. 20B, C).

However, in the context of sewage treatment, the presence of bacteria is inevitable due to environmental factors. Incorporating antibacterial properties is essential to mitigate potential health risks, such as challenges posed by bacterial contamination in SDIEs. Wang et al. [163] engineered an interconnected porous cellulose hydrogel through crosslinking hydroxypropyl cellulose with hydroxylated CNTs coatings (Fig. 20D). The evaporators demonstrate excellent purification performance (Fig. 20E) while exhibiting effective biofouling resistance under illumination (Fig. 20F). Under both dark and illuminated conditions, the antibacterial efficiency of CP/CNTs was found to be 55.1% ± 21.3% and 100%, respectively, with the enhanced antibacterial activity under light irradiation can be attributed to the combined effect of the intrinsic antibacterial properties of CP/CNTs and the light irradiation [31, 166]. The CH/CNTs exhibit consistent antibacterial activity regardless of illumination conditions, potentially attributable to residual chemical agents from the cellulose hydrogel synthesis process.

Zha et al. [5] reported a MXene-functionalized cellulose fibrous membrane with inherent anti-biofouling properties for high-performance solar desalination. The MXene/cellulose membrane exhibits 99.9% bacterial inhibition, attributable to the bacteriostatic properties of the MXene coating (Fig. 20G). After 24 h, bacterial colonization on the MXene/cellulose membrane surfaces is markedly lower than on cellulose and rGO/cellulose membranes (Fig. 20H). The condensed water from both bacterial suspensions remains transparent following photothermal purification, indicating no detectable bacterial colonies on nutrient agar plates. Water quality analysis confirms compliance with the standard of Chinese national drinking water (Fig. 20I), demonstrating the MXene/cellulose membrane's effective purification capacity and broad-spectrum antimicrobial activity against both Gram-positive and Gram-negative bacteria. These systems exemplify two antibacterial paradigms: (1) active photothermal disinfection (CNT-induced hyperthermia) and (2) passive contact biocidal action (MXene nanoblades). While photothermal approaches demand light exposure, MXene membranes function continuously, albeit with higher material costs. Future designs must balance operational energy inputs, material sustainability, and lifecycle costs for scalable deployment.

### Atmospheric Water Harvesting

Moisture in the atmosphere has been considered a rich resource for alleviating water scarcity, prompting advancements in atmospheric water harvesting (AWH) technologies (AWH) [167, 168]. A typical AWH cycle involves the absorption of vapor by hygroscopic materials (i.e., desiccants), followed by solar-driven desorption and condensation of the released vapor. Integrating these systems with interfacial evaporation techniques offers a synergistic approach to enhance freshwater production efficiency [169, 170].

**Fig. 21:**
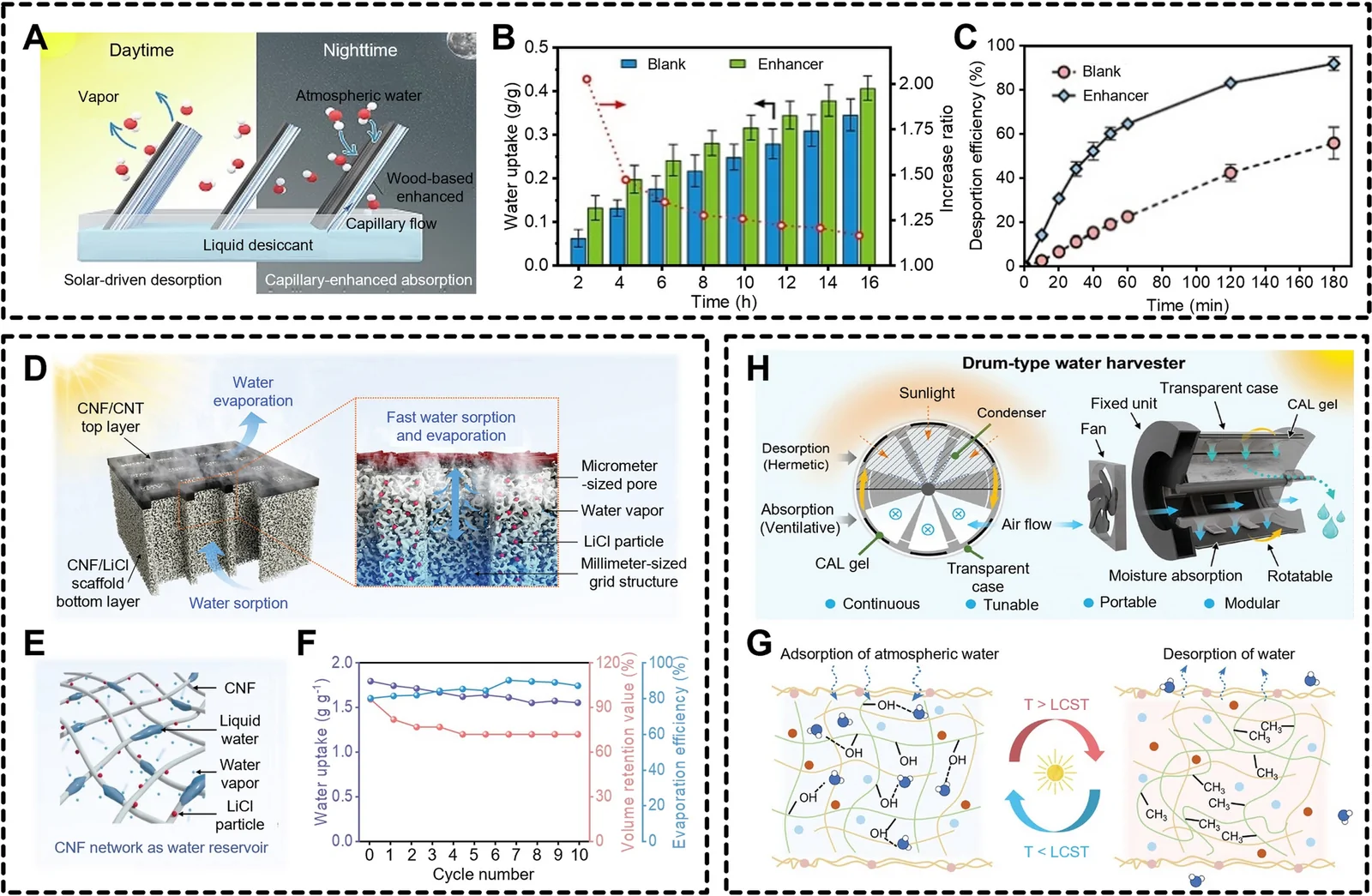
**A** Diagram illustrating the configuration of a solar-driven atmospheric water harvesting (AWH) device enhanced by a wood-based material. **B** Water absorption capacity of the sample, with and without wood-based enhancers, over a 16 h period. **C** Comparison of desorption efficiency in the desiccant, in the presence and absence of wood enhancers [171]. **D** Schematic diagrams illustrating the architecture and working mechanism of the bilayer scaffold sorbents fabricated through 3D printing. **E** Illustrative schematic showing the CNF scaffold functioning as a water reservoir for storing absorbed water. **F** The performance of bilayer scaffold-1.5 in terms of water uptake, volume retention, and evaporation efficiency was measured during cyclic testing. D-F) [13]. **G** Cellulose/alginate/lignin (CAL) gel design concept includes moisture adsorption and desorption, at low and high temperature, respectively. **H** Schematic of a solar-driven and drum-type harvester that operate continuously with multiple adsorption–desorption cycles [173]

Recent innovations in hygroscopic material design highlight the potential of bio-derived architectures. Deng et al. [171] engineered a photothermal wood-based enhancer through partial delignification and unilateral low-temperature carbonization (Fig. 21A). The LiCl/TEG desiccant mixture reduces the saturated vapor pressure of the solution through strong water–molecule interactions. Moisture absorption is enhanced by increasing the gas–liquid interfacial area through capillary-driven transport and spreading of the liquid desiccant on the enhancer surface [172]. The enhanced system achieved a moisture absorption rate of 0.137 g g^−1^ in 2 h at 60% relative humidity, exceeding that of the nonenhanced sample by more than a factor of two (Fig. 21B). Solar-driven desorption yielded a rate of 1.190 kg m⁻^2^ h⁻^1^, doubling the efficiency of conventional setups (Fig. 21C).

Similarly, Zhu et al. [13] engineered a bilayer scaffold via 3D printing, incorporating CNF, LiCl, and CNTs into an integrated structure (Fig. 21D). The 3D-printed and freeze-dried multiscale porous base layer, assisted by LiCl, enables atmospheric water vapor capture. Subsequent water diffusion into the CNF scaffold's internal structure facilitates storage within its hydrophilic network (Fig. 21E). The CNF/CNT top layer efficiently converts solar energy to thermal energy for water evaporation. Hydrophilic CNF networks facilitated internal water storage, maintaining 80–90% evaporation efficiency over 10 cycles with minimal structural degradation (< 30% shrinkage) (Fig. 21F).

While most AWH devices rely on single-compartment designs for sequential adsorption–desorption cycles, which are optimal for materials with high capacity but slow kinetics. However, Zhou et al. [173] reported a bio-based gel (CAL gel) with a rapid adsorption–desorption rate. The moisture adsorption mechanism of CAL gel includes several stages: (1) During adsorption, LiCl—uniformly distributed on the surface and within voids of the CAL gel—captures water molecules by forming crystalline water compounds, followed by diffusion into the polymer network (SA/HPC), where hydroxyl groups mediate efficient water transport. (2) The gel achieves desorption easily through sunlight-induced evaporation. (3) During the desorption phase, when the temperature exceeds the LCST, the HPC component undergoes a transition from hydrophilic to hydrophobic, causing hydrogen bonds in HPC to dissociate and form hydrophobic –CH_3_ groups, further facilitating water desorption (Fig. 21G). On the basis of the merits of CAL gel designed a solar-driven, drum-type, tunable, and portable harvester that can harvest atmospheric water within a brief time (Fig. 21H). The device features dual chambers: a lower compartment for ambient moisture adsorption (enhanced by an integrated fan) and an upper sealed chamber for solar-driven desorption. A 180° rotation repositions the CAL gel between chambers, enabling continuous cyclic operation with minimal energy input.

### Collaborative Photocatalytic Hydrogen Production

Photocatalytic water splitting for hydrogen production pioneers a promising approach for converting solar energy into green energy. However, the wide bandgap of conventional photocatalysts and the limited presence of UV photons in solar radiation result in the majority of low-energy visible and near-infrared light being absorbed and converted into heat. Integrating SDIE with photocatalysis offers a synergistic solution, coupling thermal energy generation with hydrogen production.

**Fig. 22:**
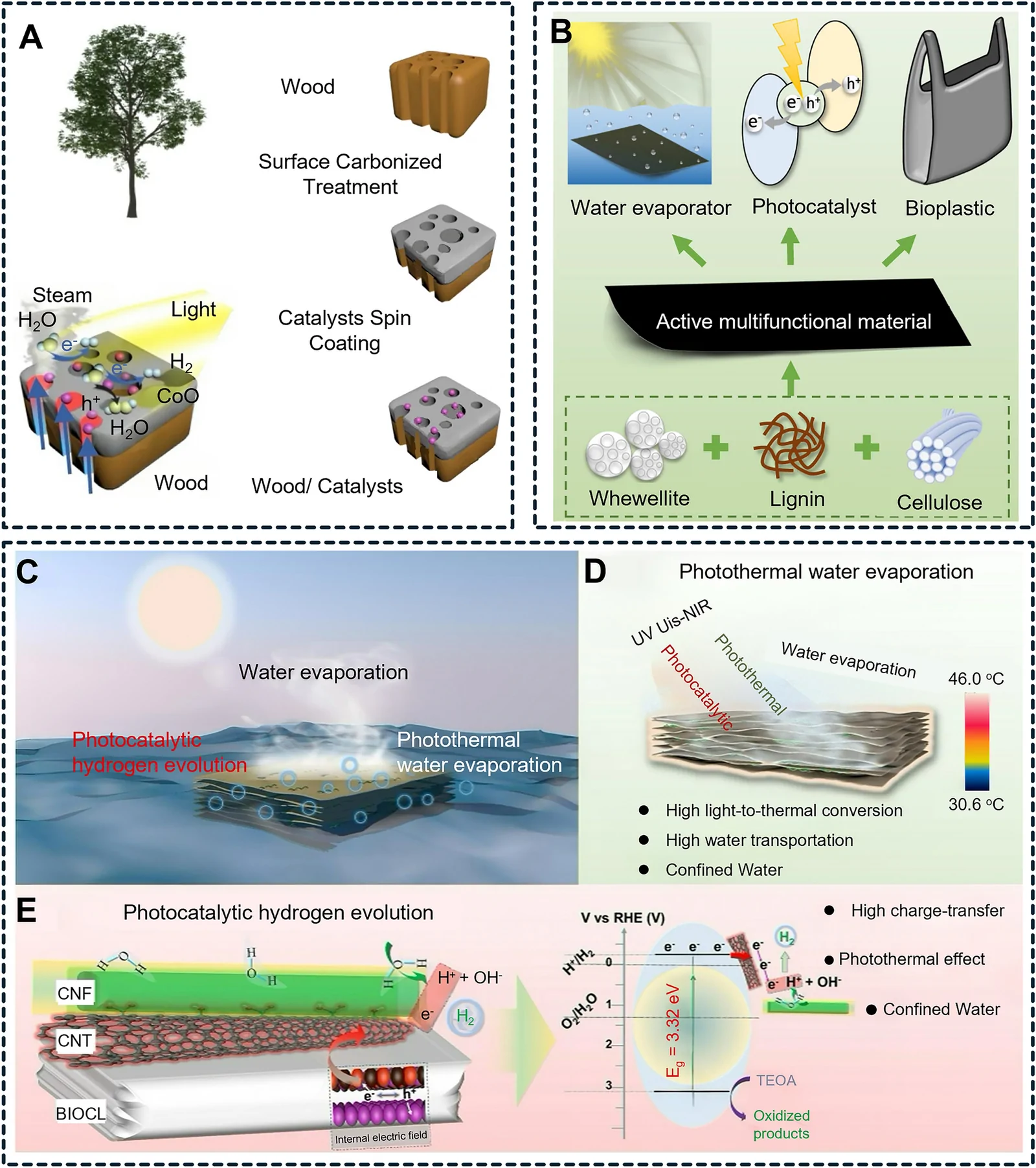
**A** Fabrication procedure for the wood/photocatalyst architecture, which both generates water steam and catalyzes its splitting to drive hydrogen evolution, is schematically depicted [174]. **B** Schematic of the preparation and application scenario of the active multifunctional material (AMM) [175]. **C**–**E** Schematics of the mechanism of photothermal water evaporation (PWE) and photocatalytic hydrogen evolution (PHE) in the interfacial evaporation system [176]

Recent breakthroughs in hybrid systems underscore this potential. Guo et al. [174] developed an efficient photo-thermal catalytic system that utilizes charred wood substrates to convert liquid water into steam and produce hydrogen under solar illumination without auxiliary energy (Fig. 22A). This system enhances hydrogen transport kinetics while lowering interfacial energy barriers for water adsorption, achieving a remarkable hydrogen evolution rate of 220.74 μmol h⁻^1^ cm⁻^2^. In parallel, Fang et al. [175] synthesized an amorphous mineral matrix (AMM) by integrating whewellite with lignin and cellulose, derived from red maple leaves (Fig. 22B). The heterostructure of this composite facilitates broadband solar absorption and efficient charge separation, yielding dual functionality in solar evaporation and photocatalytic hydrogen generation. In addition, Zhou et al. [176] simultaneously achieved green energy generation and freshwater supply by developing a dual-functional 2D layered membrane (2DLM) composed of BiOCl nanosheets, CNFs, and CNTs (Fig. 22C). The 2DLM harnesses localized photothermal heating to drive water evaporation (2.05 kg m⁻^2^ h⁻^1^) while leveraging CNTs as conductive pathways to enhance charge separation. This dual mechanism reduces interfacial resistance and carrier recombination, achieving a hydrogen production rate of 22.64 μmol g⁻^1^ h⁻^1^. Such systems exemplify the synergy between photothermal activation and photocatalytic efficiency, where electron-lattice collisions elevate local temperatures to activate reactants, amplifying catalytic performance (Fig. 22D, E).

## Conclusion and Perspective

**Fig. 23:**
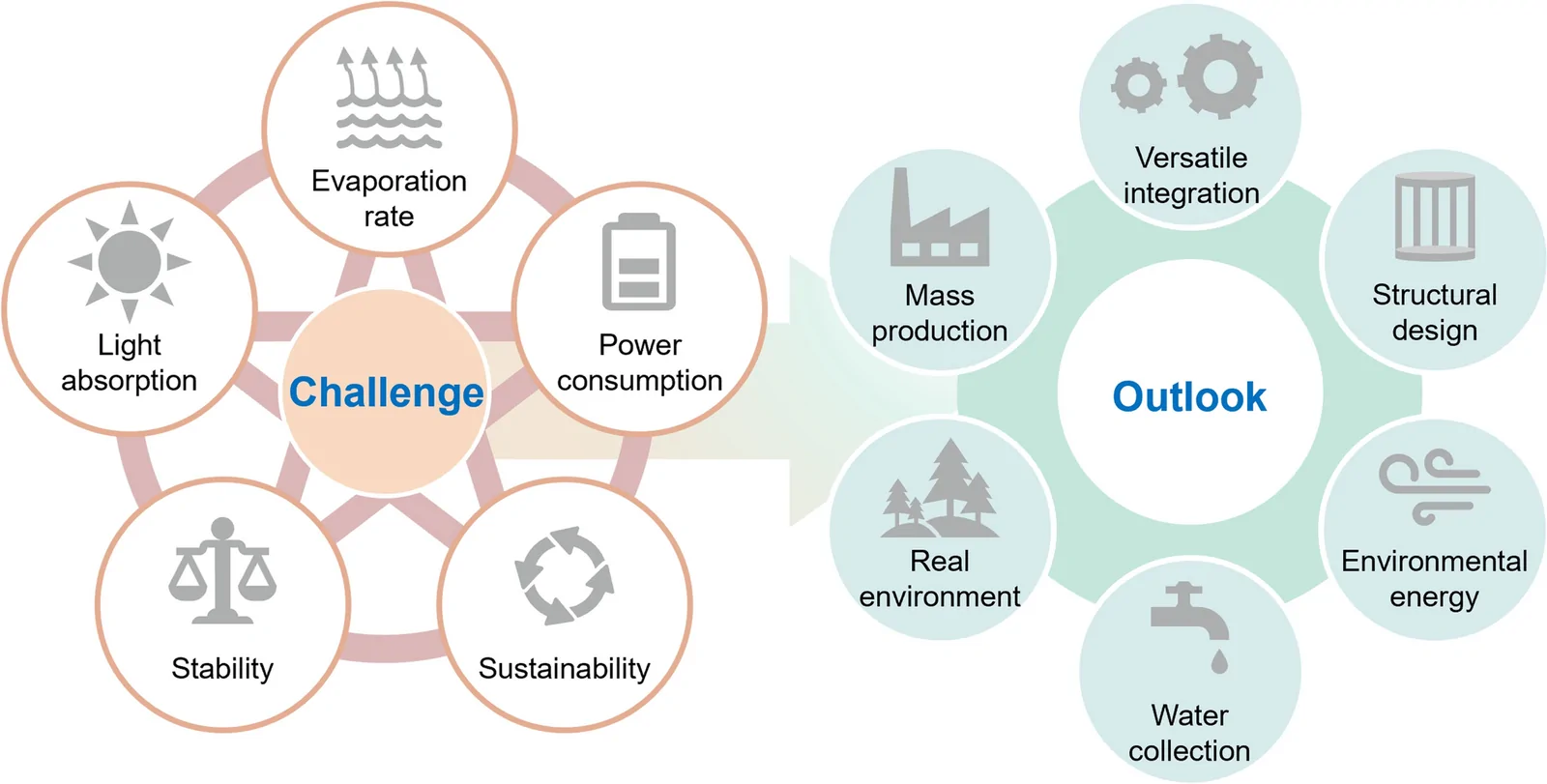
Challenge and outlook of lignocellulosic biomass-based materials for SDIEs

Solar energy stands as a pivotal renewable resource in addressing the dual challenges of global energy security and freshwater scarcity. SDIEs have emerged as a sustainable solution, utilizing solar radiation to passively generate clean water through an environmentally benign process. Lignocellulosic biomass, with its natural renewability and unique physicochemical properties, offers a versatile and sustainable platform for advancing next-generation SDIEs technologies. In this review, we examine the relationship between lignocellulosic biomass and SDIEs, highlighting key design strategies for optimizing their performance. We further discuss advanced hydro-thermal management approaches to enhance evaporation efficiency, enabling scalable and sustainable clean water production. Additionally, we explore the potential for multifunctional integration, providing a roadmap for future innovations in lignocellulosic biomass-based SDIEs. While the advantages of these materials have been extensively demonstrated, several key challenges must still be addressed before they can be widely adopted in practical applications (Fig. 23).

First, lignocellulosic biomass-based SDIEs exhibit limited evaporation rates under practical operational conditions. This limitation stems from two fundamental challenges: insufficient reduction of the evaporation enthalpy of water and substantial thermal losses through conduction and convection. These inefficiencies are intrinsically linked to the hierarchical microstructure of lignocellulosic biomass-based materials, characterized by anisotropic pore distributions, heterogeneous porosity, and high tortuosity—features that simultaneously mediate water transport and thermal regulation. While structural modifications and advanced material designs show promise for performance enhancement, the fundamental mechanisms governing heat and mass transfer in these complex natural systems remain incompletely understood.

Second, conventional production methods, including high-temperature carbonization and freeze-drying, demand substantial energy inputs. While these processes enhance material properties critical for solar desalination performance, their reliance on energy-intensive technologies undermines the overall sustainability of SDIEs by increasing carbon footprints and manufacturing costs. This trade-off between performance enhancement and energy consumption creates a critical barrier to scaling economically viable and environmentally sustainable solar desalination technologies.

The third layer of complexity arises during the modification and functionalization of lignocellulosic biomass composites. Chemical treatments and synthetic additives, though effective in tailoring material properties, introduce environmental risks across the material lifecycle. Toxic residues from chemical processes may persist during production, use, and eventual degradation, while the energy and resource demands of these modifications further erode the inherent sustainability advantages of biomass-derived materials. Consequently, the central challenge lies in reconciling performance optimization with environmental stewardship: How can we engineer high-efficiency biomass composites without perpetuating energy-intensive practices or introducing hazardous substances.

Addressing these interconnected issues necessitates a paradigm shift toward green chemistry principles. Low-energy modification techniques and nontoxic additives must replace conventional high-impact methods. Simultaneously, process innovation—such as one-step conversion for the economical and green preparation of graphene oxide on a gram scale from biomass at room temperature under atmospheric pressure—could mitigate energy and resource burdens [177]. By prioritizing such strategies, the field can advance SDIEs that fulfill both technical and sustainability criteria, ensuring their viability as scalable solutions for global water scarcity challenges.

Fourth, outdoor durability is compromised by microbial degradation and environmental exposure. Lignocellulosic biomass based can serve as a carbon source for a variety of microorganisms, including fungi, bacteria, and protozoa, which contribute to its degradation when exposed to aquatic environments. This microbial activity accelerates the deterioration of the material, ultimately compromising the efficiency of the evaporation process. To address these challenges, a multifaceted approach is required. Optimization of the production processes to reduce energy consumption is one avenue, while the application of protective coatings or treatments may enhance the resistance of materials to microbial degradation. Additionally, the development of alternative, more durable materials or hybrid systems could further improve the longevity and performance of SDIEs, ensuring their viability as a sustainable solution for water purification in the long term.

Fifth, lignin exhibits inherent photothermal conversion capabilities, yet its application in SDIEs has been limited by its relatively low light absorption efficiency. This issue has prompted a concerted effort among researchers to enhance light absorption properties of lignin, thereby unlocking its potential as a viable photothermal material in solar-driven systems. Lignin structure contains abundant UV-absorbing chromophores, such as conjugated phenols, ketones, quinones, and intramolecular hydrogen bonds, which facilitate its absorption of ultraviolet light. However, this narrow absorption spectrum constrains its broader application in photothermal systems. Recent advancements suggest that photothermal performance of lignin can be significantly improved through structural modifications that increase its *π*–*π* conjugation and stacking, as well as through physical processing techniques. While these advancements mark significant progress in enhancing photothermal properties of lignin, the relationship between structural characteristics of lignin—such as the nature and concentration of functional groups, molecular weight, and lignin subclass—and its photothermal behavior remains inadequately understood. Further investigations into these structural factors, as well as more comprehensive studies on the use of lignin in SDIEs, are necessary to fully harness its potential as a sustainable photothermal material. In the field of interfacial evaporators, lignin holds promise to become a “photothermal nova” in the future.

Although these challenges present significant barriers to practical implementation, they also reveal critical opportunities for advancing next-generation lignocellulosic biomass-based SDIEs. In recent years, lignocellulosic biomass-based SDIEs have undergone significant advancements, broadening their applications far beyond the traditional scope of seawater desalination. A notable trend in this evolution is the shift toward multifunctionality, where modern lignocellulosic biomass-based SDIE designs increasingly incorporate a variety of additional capabilities. These include salt recovery, wastewater treatment, antibacterial properties, catalysis, and energy generation. The integration of such functionalities has not only enhanced the versatility of lignocellulosic biomass-based SDIEs but also expanded their potential applications into diverse fields, including energy and environmental management. For instance, Su et al. achieved concurrent seawater desalination, radiative cooling, and uranium extraction [178]. Li et al. and Zhu et al. demonstrated systems for simultaneous freshwater and critical element recovery, such as boron [179] or cesium [180], respectively. Furthermore, Lin et al. created an innovative self-rotating evaporator capable of treating challenging oily saline wastewater [181]. This multifaceted design opens new avenues for lignocellulosic biomass-based SDIEs in industrial and technological domains, positioning them as essential components in addressing global challenges in water, energy, and environmental sustainability.

Building on this trend, recent innovations in SDIE design have focused on integrating renewable environmental energy sources, further enhancing their multifunctionality. Among the most promising developments is the utilization of ambient energy, which has emerged as a key area of exploration. For instance, interfacial evaporators modeled after waterwheels capture tidal energy [182], while those inspired by windmills harness wind energy [183]. These pioneering approaches not only expand the range of renewable energy applications but also hold great potential for sustainable water desalination and other industrial processes. As research in this domain progresses, the integration of energy-harvesting technologies into lignocellulosic biomass-based SDIEs is poised to further optimize their efficiency and sustainability, potentially reshaping the future of resource-efficient evaporation systems.

At the core of SDIE operation is the efficient collection of freshwater through evaporation. However, evaporation performance alone does not fully capture desalination efficiency, as the incorporation of a condenser into the system can significantly reduce the evaporation rate. The condensation process, whereby water droplets accumulate on the surface of condenser, leads to light reflection and scattering, which further impairs evaporation efficiency. While many existing lignocellulosic biomass-based SDIEs achieve high evaporation rates, relatively few have incorporated innovative condensation systems designed to enhance freshwater collection without compromising evaporation performance. Therefore, future research should focus on integrating advanced condensation mechanisms into lignocellulosic biomass-based SDIEs to enable rapid freshwater collection while maintaining high evaporation efficiency. A prominent solution is the inverted-structured system, which ingeniously channels vapor downward to exploit the thermal gradient between generated steam and cooler feed water or ambient conditions. This configuration has demonstrated remarkable performance, achieving a daily freshwater yield of 13.68 kg m^−2^ under natural sunlight—an efficiency improvement of 119% over traditional designs [184, 185]. Parallelly, the multi-stage solar still concept achieves thermodynamic superiority through latent heat recycling, where vapor condensation energy is repurposed to drive subsequent evaporation stages. Empirical studies validate the scalability of this approach, showing evaporation rates rising from 0.74 kg m^−2^ h^−1^ in single-stage systems to 1.84 kg m^−2^ h^−1^ in six-stage configurations [186]. These complementary strategies represent transformative advances in solar-driven desalination, establishing new paradigms for high-yield freshwater production.

The translation of lignocellulosic biomass-based SDIEs from promising laboratory prototypes to mass production presents a critical yet achievable frontier. While challenges in scaling remain, the intrinsic advantages of these materials—including their environmental sustainability and natural abundance—provide a compelling foundation for development. To scale lignocellulosic biomass-based SDIEs from laboratory settings to industrialization scale, a concerted focus on several key areas is imperative. First, the standardization and green pretreatment of feedstocks are essential to ensure consistent performance and cost-effectiveness. Second, manufacturing must evolve from batch processes to continuous, low-energy workflows to enable large-scale production. From a design perspective, integrating long-term stability, anti-fouling properties, and modular architectures is crucial for reliable system integration and field maintenance. Ultimately, the technology viability as a freshwater solution must be validated through comprehensive cost and life-cycle assessments, confirming its economic competitiveness and net environmental benefit. Therefore, continued research and exploration are indispensable, not only to refine the material and structural design of SDIEs but also to systematically address these fundamentals, thereby overcoming the barriers to their large-scale deployment.
